# Contemporary accounts and future directions for decolonising global health: A scoping review

**DOI:** 10.1016/j.puhip.2026.100874

**Published:** 2026-10-12

**Authors:** Daniel E. Stewart, Tiruneh Amsalu, Ellie Fairfoot, David Keen, Jessica Keenan, Frances Butcher, Kevin Miles, Ahmed Razavi

**Affiliations:** a International Health Regulations Strengthening project, Global Operations, UK Health Security Agency, London, UK; b Africa Centres for Disease Control and Prevention, Addis Ababa, Ethiopia; c UK Public Health Rapid Support Team, UK Health Security Agency, UK; d Global Health Unit, NHS England, London, UK; e Workforce, Training and Education, NHS England, London, UK; f The Ethox Centre, Nuffield Department of Population Health, University of Oxford, Oxford, UK

**Keywords:** Decolonising global health, epistemic injustice, equity

## Abstract

**Objectives:**

Though much has been written about the importance of decolonising global health, there is a lack of consensus around how it should be defined, conceptualised and actioned, and who has responsibility to do so. In accordance with PRISMA guidelines, we undertook a scoping review of the decolonising global health literature in order to explore the meaning of decolonising global health, to identify examples of best practice, and to find out how those writing about the issue see the future of the movement.

**Study design:**

Scoping review.

**Methods:**

We searched databases (Embase, EBSCO Global Health, Medline and Scopus) for peer-reviewed and grey literature with titles and abstracts, and then full texts double-screened by authors to identify papers for inclusion. Our search strategy focussed on opinions and discourse using terms broadly linked to decolonising global health. Data, including conclusions and recommendations, were extracted and results presented as a narrative synthesis of included papers to provide a contemporary account of the decolonising global health agenda.

**Results:**

Included papers (n = 129) were predominantly commentary or opinion pieces (n = 95). Authors of the included papers were affiliated with institutions predominantly from high income countries including the USA (n = 53) and UK (n = 30). Included papers presented structures and of definitions for decolonising global health, described the historical, colonial influence on global health, explored power imbalances in current global health structures, and made a number of suggestions as to how to address these imbalances.

**Conclusions:**

Despite the clear imperative in the literature to take action, there is no clear consensus on where to start. Drawing from the findings of our review, we conclude with a set of recommended approaches and next steps for decolonising global health, focussing on epistemic justice, partnership working, the structure of global health, and individual duty.

**What this study adds**

• This study addresses a significant gap in the literature by providing a synthesis of the definitions, historical legacies, and key themes of the decolonising global health movement across 129 peer-reviewed and grey literature sources.

**Implications for practice**

• The study moves beyond theoretical critique to propose a set of recommended approaches for global health actors, focussed on addressing epistemic injustice, fostering equitable partnership working, reforming global health structures, and encouraging individual duty.

## 1 Introduction

Global health actors and institutions are expected to hold the principles of health equity and justice at the centre of what they set out to achieve. Foundational policy frameworks reinforce this expectation: the WHO Constitution, adopted in 1946, describes health as a fundamental right of ‘every human being’ [1]. The Ottawa Charter in 1986 explicitly identifies "social justice and equity" as a prerequisite condition for health [2]. Yet, contemporary practice routinely falls short of these ideals. The international response to the COVID-19 pandemic, to a large extent, reinforced pre-existing structural inequities and injustices. This was most visibly demonstrated by disparities in access to vaccines, personal protective equipment, and medical treatments globally, alongside the power imbalances evident in international research partnerships [3], [4], [5].

A key injustice relates to the continuing legacy of our colonial past. The decolonising global health agenda recognises that the way in which global health is taught, practiced and implemented often perpetuates historically rooted and exploitative power structures, and seeks to address this [6], [7]. Specifically, it seeks to dismantle these power structures by moving beyond representational diversity to shift resource control, research agendas, and leadership to local communities and institutions [8], [9].

However, despite many of those working within global health calling for decolonisation, there is a lack of consensus on how decolonising global health should be defined, conceptualised, or operationalised. Current discourse remains fragmented, largely theoretical, and dominated by authors affiliated with high-income country (HIC) institutions.

Whilst much has been written about the importance of decolonising global health in the peer-reviewed and grey literature, to our knowledge, previous scoping or systematic reviews have been limited in scope or focussed on specific niches such as decolonising global health evaluation [10], or gender in decolonial discourses [11].

This review responds to a gap in the literature by providing a broad-based synthesis of the decolonising health movement across the peer-reviewed and grey literature. By exploring viewpoints across the field, this review seeks to understand perspectives on decolonising global health, summarise viewpoints and key themes arising from both the conceptual and empirical literature, and to generate a pragmatic set of actions for global health actors and institutions looking to adopt a decolonising approach.

Our specific aims were to.

a. Collate and critically appraise publications focussed on decolonising global health;
b. Explore themes within the literature, including the conceptualisation and definitions of decolonising global health, examples of best practice actions to decolonise global health, and the challenges to and opportunities for action on this agenda;
c. Generate a set of proposals for people working in global health research, programme implementation and policy with a view to direct future action focussed on decolonising global health.

## 2 Methods

We undertook a scoping review of the literature in accordance with PRISMA guidelines for scoping reviews [12] and guided by the methodological framework proposed by Arksey and O'Malley [13]. Scoping reviews are well suited to providing an overview of evidence and for clarifying concepts and definitions [14]. The protocol for this study, registered prior to its undertaking, can be found here: https://osf.io/ynpkf/

### 2.1 Definitions

For the purposes of this paper, we have defined key terms as follows:

**Decolonisation:** the process of freeing individuals, communities, institutions and spheres of activity (e.g. education and health systems) from the broader economic, social, cultural and psychological aspects of the colonial experience.

**Equity:** fairness and justice in the way people are treated recognising that we do not all start from the same place.

**Anti-racism:** the practice of actively identifying all forms of racial inequity and actively finding ways to oppose and/or eliminate these.

**Colonialism:** One country extending political, economic, cultural control over another country or territory.

**Coloniality:** Patterns of power that emerged as a result of colonialism but have continued beyond colonialism.

**Global health:** Study, research and practice that is focused on improving health and health equity for all people worldwide, with a focus on transnational health challenges.

### 2.2 Eligibility criteria and search strategy

Any study or paper type was included in our review including publications both in the peer-reviewed and grey literature. No date restrictions on publication date were applied. Papers published in the English language only were included, a pragmatic choice determined by project resources and access to translation services.

We searched Embase, EBSCO Global Health, Medline and Scopus bibliographic databases. A grey literature search using Google search and Google Scholar was also undertaken to capture literature and broader perspectives that may not be indexed by the bibliographic databases. In addition to database searches, we also undertook citation searches using the R package citationchaser [15], using seven ‘pearl’ papers which we identified from an initial search and through our own reading [16], [17], [18], [19], [20], [21], [22], [23], [24]. Citation chasing across a targeted set of foundational papers ensured key literature was captured while avoiding unnecessary redundancy across all included articles. To ensure the literature we included was as up to date as possible, we undertook searches at two points, the first in September 2022 and the second in December 2024.

Papers which directly addressed or explored decolonising global health, including discussions around how it is defined, conceptualised and put into practice were eligible for inclusion.

Because ‘decolonising global health’ is not indexed as a standardised term in the bibliographic databases we used, we adapted our search strategy (S1 Appendix) to the literature to capture the breadth of the literature, both empirical and theoretical. We used a broad set of terms including global health, colonialism, racism, epistemic injustice, transnational partnerships and intersectionality, in addition to a system filter for letters and editorials. A term filter in the form of a search string was also used to narrow the results. All terms were searched in abstracts, keywords, subject headings, titles and text words.

Full details of the search strategy, search terms and number of results returned is available in the supplementary material.

### 2.3 Screening

Search results were de-duplicated using Endnote prior to being screened. The large volume of resulting articles was divided amongst the following authors (DS, TA, EF, DK, JK, KM, and AR). Each title and abstract were independently screened by a minimum of two of the above authors using the Rayyan review screening tool [25] and those records which clearly did not meet inclusion criteria were excluded. To ensure inter-rater reliability, the authorship team held sessions to review a few examples of papers and discussing inclusion and exclusion criteria amongst the group to develop a shared understanding. Two authors then independently screened full text articles for inclusion or exclusion. Discrepancies or disagreements were addressed first through discussion and if needed by referral to a third author.

### 2.4 Data extraction

Data was extracted into a co-designed and pre-agreed Microsoft Excel form. Data extracted included author, institutional affiliation, publication date, and geographic focus. Extracted key conclusions captured attempts to define, conceptualise, and critique decolonising global health, while recommendations encompassed specific frameworks, policy proposals, and operational solutions. Data were extracted independently for each paper by a minimum of two authors with disparities and disagreements discussed first and referred to a third author if necessary.

### 2.5 Data synthesis

The approach to data synthesis was guided by the aims of the study and the need to robustly present results that included both a high proportion of commentary and opinion-based articles. The data extraction template was designed to be sufficiently flexible to allow the authors to populate relevant findings from a large variety of studies. As a consequence of this, no articles were excluded on the basis of quality. This was because the aim of the scoping review was to collate the full range of what has been written about decolonising global health. A risk with applying a quality assessment tool to this literature is that it may inadvertently perpetuate the same biases that the decolonising agenda seeks to address. Khan et al. [17], argue that the decolonisation of academic publishing spaces needs to make room for publications from alternate epistemic standpoints and de-emphasise traditional hierarchies of evidence and practice. This review therefore seeks to meet this aspiration, whilst acknowledging the limitation that the focus on traditional academic literature may also reinforce these biases as consideration of videos, storytelling, and knowledge produced in other languages was not able to be included.

Instead, the authors worked together to undertake a narrative synthesis of the findings in all the included articles, through summarising the key conclusions and recommendations of each article and then compiling them into themed topic areas, to provide a contemporary account of the decolonising global health agenda. Our narrative approach was informed by established guidance for narrative synthesis [26] alongside standards for meta-narrative reviews [27]. Narrative synthesis has been shown to be a useful technique in synthesising different types of studies without losing the diversity in study designs and contexts [27]. In this study, the Mindmeister tool was used [28] to discuss, review, map out and agree key themes.

The findings from the included articles are summarised below. The narrative synthesis first examines conceptual perspectives, encompassing author demographics, core definitions, historical legacies, and rationales for reform. It then synthesises actionable recommendations across epistemic, partnership, and governance domains, before evaluating implementation barriers and low and middle-income country (LMIC) author perspectives. The discussion then analyses this literature to consider gaps, opportunities and directions for future research.

### 2.6 Positionality and reflexivity statement

The authors of this paper comprise health practitioners and researchers primarily affiliated with, and writing from the gaze of HICs [29]. We are actively engaged in the implementation of global health projects in LMICs, seeking to better understand how we can decolonise the implementation of our work. Our positionality within HIC institutional frameworks inherently shapes our perspectives, the research questions we asked throughout this review, and has determined the sources of knowledge we were able to access, and our interpretation of the findings. Reflecting on our reliance on English-language sources is one element of this positionality, illustrating how our own research processes remain bound to the broader structural coloniality of knowledge production.

## 3 Results

Electronic database searches identified 2389 records with a further 263 records included through citation searches and further reading (Table 1). Following de-duplication, title and abstract and full-text screening, 129 articles were included in our review (Fig. 1: PRISMA flow chart).

**Table 1:** Summary of all papers included in the review.

Title	Year	Authors	Country	Publication Name	Type of publication	Type of article	Key themes
4 Ways to Decolonise Global Health	2022	Joanne Silberner	USA	Hopkins Bloomberg Health Magazine	Grey literature	Commentary/Opinion piece	Colonial origins of global health, medical education, reflective practice, individual duty
A proposed guide to reducing bias and improving assessments of decolonisation in global health research	2024	Christine Ngaruiya, Muzzammil Imran Muhammad, Nadia A. Sam-Agudu	USA, Nigeria, Ghana	Frontiers in Education	Journal	Commentary/opinion piece	Moving beyond rhetoric to decolonise global health research, structural change, domains for decolonising global health, power dynamics in global health research, decolonising as an iterative process.
A toolkit for decolonizing global emergency medicine education	2023	Adeline Dozois, Catalina González Marqués, Kaushila Thilakasiri, Adebisi Anthonia Adeyeye, Joseph Leanza, Megan Rybarczyk, Timothy Depp, Travis Wieland, Naz Karim, Monalisa Muchatuta, Fahad Ali, Ahmed Amer, Stephanie Chow Garbern, Shama Patel	USA, Sri Lanka, Nigeria,	Frontiers in Education	Journal	Mixed methods study	Decolonising global emergency medicine, barriers to global emergency medicine education, cultural humility.
Accountability framework to decolonise global health	2021	Bolajoko O. Olusanya	Nigeria, South Africa	The Lancet	Journal	Commentary/Opinion piece	Need for systems and frameworks to hold global health institutions accountable
Actions for decolonizing global health	2022	Garry Aslanyan, Catherine Kyobutungi, Agnes Binagwaho	Canada, Uganda, Rwanda	TDR Global Matters Podcast (transcript)	Grey literature	Commentary/Opinion piece	White supremacy, models of finding for global health, systems change, global health academia
Addressing power asymmetries in global health: Imperatives in the wake of the COVID-19 pandemic	2021	Seye Abimbola, Sumegha Asthana, Cristian Montenegro, Renzo R. Guinto, Desmond Tanko Jumbam, Lance Louskieter, Kenneth Munge Kabubei, Shehnaz Munshi, Kui Muraya, Fredros Okumu, Senjuti Saha, Deepika Saluja, Madhukar Pai	Australia, India, Chile, Philippines, Ghana, South Africa, Kenya, Tanzania, Bangladesh, Canada	PLOS Medicine	Journal	Commentary/Opinion piece	Power, resource and knowledge inequities in global health
Anti-Racism and Anti-Colonialism Praxis in Global Health-Reflection and Action for Practitioners in US Academic Medical Centres	2021	Zeinabou Niamé Daffe, Yodeline Guillaume and Louise C Ivers	USA	The American Society of Tropical Medicine and Hygiene	Journal	Commentary/Opinion piece	Reflective practice as a tool in decolonising global health, anti-colonialism, anti-racism
Applying a Power Analysis to Everything We Do: A Qualitative Inquiry to Decolonise the Global Health and Development Project Cycle	2023	Doreen Tuhebwe, Sarah Brittingham, Amandari Kanagaratnam, Elikem Togo, Funmilola M. OlaOlorun, Rhoda K. Wanyenze, Ndola Prata, Allysha C. Maragh-Bassb	Uganda, USA, Nigeria	Global Health: Science and Practice	Journal	Qualitative study/Literature Review	The colonial influence throughout global health project cycles, need for more equitable partnership working, redistribution of power to Global South, need for individual action and structural change.
Arts-based methods as a critical and decolonising process in global mental health: Reflections on popular discourse, artistic rigour and limitations	2024	Matthew Elliott	UK	Methods in Psychology	Journal	Qualitative study	Global mental health, masculinities and mental health, possibilities of arts-based research in decolonising mental health practices
Barriers to decolonising educational partnerships in global health	2021	John Kulesa, Nana Afua Brantuo	USA	BMJ Global Health	Journal	Literature review	Global health education, the colonial roots of global health education, equitable partnerships, decolonising health education partnerships
Beyond pledges: academic journals in high-income countries can do more to decolonise global health	2021	Bolajoko O Olusanya, Macpherson Mallewa, Felix Akpojene Ogbo	Nigeria, Malawi, Australia	BMJ Global Health	Journal	Commentary/Opinion piece	Colonial history and influence on global health, epistemic injustice, call on academic journals to promote equity, equality, diversity and inclusiveness principles related to authorship
Build that wall! Vaccine certificates, passes and passports, the distribution of harms and decolonial global health justice	2021	Gabriela Arguedas-Ramírez	Costa Rica	Journal of Global Ethics	Journal	Commentary/Opinion piece	Colonial influence on global health, decolonisation as a process, viewing the COVID-19 pandemic through a decolonial lens, ethical considerations of vaccine passports
Building a framework to decolonise global emergency medicine	2023	Monalisa Muchatuta, Shama Patel, Catalina Gonzalez Marquez, Kaushila Thilakasiri, Sreenidhi Vanyaa Manian, Jennifer Chan, Ngassa Mssika, Taryn Clark, Taylor Burkholder, Nikkole Turgeon, Vinay N Kampalath, Nivedita Poola, O Agatha Offorjebe, Adeline Dozois, Gimbo Hyuha, Oluwarotimi Vaughan-Ogunlusi, Carol McCammon, Katie Wells, Megan Rybarczk, Maria Paula Castillo, Adebisi Anthonia Adeyeye, Chris A Rees, Sanjukta Dutta, Stephanie Chow Garbern	USA, Sri Lanka, India, Tanzania, Nigeria	American Emergency Medicine Education and Training	Journal	Literature review	Colonial issues associated with emergency medicine, a path for change, the disproportionate influence of faculty and students from high-income countries (HICs) in research, academic pursuits, and instruction in global health creating one-way flow of knowledge, training opportunities, funding and funding priorities from HICs to in low and middle income countries (LMICs).
Can schools of global public health dismantle colonial legacies?	2020	Ngozi A Erondu, Dorothy Peprah, MIshal S Khan	USA, UK, Pakistan	Nature Medicine	Journal	Commentary/Opinion piece	Neo-colonial academic practice, epistemic injustice, representation in academic leadership, anti-racism at academic institutions
Cancer linguistics and the politics of decolonizing health communication in Coastal Tanzania: Reflections from an anthropological investigation	2024	Daniel W. Krugman, Athumani Litunu, Saumu Mbeya, M. Yunus Rafiq	USA, Tanzania, China	Social Science & Medicine	Journal	Commentary/Opinion piece	The construction of biomedical language and the role of language in decolonising global health, the need to dismantle incommunicability and the global order that supports it.
Challenges for breaking down the old colonial order in global health research: the role of research funding	2021	Larissa Fortunato Araújo, Flávia B Pilecco, Francisco Gustavo Silveira Correia, Marcelo José Monteiro Ferreira	Brazil	The Lancet Global Health	Journal	Commentary/Opinion piece	Inequities in the funding perpetuating colonial structures and views within global health
Colonialism, malaria, and the decolonisation of global health	2022	Jesse B Bump, Ifeyinwa Aniebo	USA, Norway, Nigeria	PLOS Global Public Health	Journal	Literature review	Malaria control, colonisation, tropical medicine
Committing to anti-racism reforms? Three critical building blocks for global health organisations	2022	Mishal S Khan, Angela Obasi, Rinki Deb, Serign Jawo Ceesay	United Kingdom	PLOS Global Public Health	Journal	Commentary/Opinion piece	Anti-racism, academia, institutional racism, decolonising global health curricula, research
Community Engagement in Cutaneous Leishmaniasis Research in Brazil, Ethiopia, and Sri Lanka: A Decolonial Approach for Global Health	2022	Kay Polidano, Linda Parton, Suneth B Agampodi, Thilini C Agampodi, Binega H Haileselassie, Jayasundara M G Lalani, Clarice Mota, Helen P Price, Steffane Rodrigues, Getachew R Tafere, Leny A B Trad, Zenawi Zerihun, Lisa Dikomitis	UK, Sri Lanka, Ethiopia, Brazil	Frontiers in Public Health	Journal	Qualitative study	Community engagement, Eurocentric knowledge production in global health, colonial legacies within global health, deconstructing dominant western approaches in global health practice
Community First solutions for COVID-19: decolonising health crises responses	2021	Rachel Kiddell-Monroe, Jessica Farber, Carol Devine, James Orbinski	Canada	The Lancet Planetary health	Journal	Commentary/Opinion piece	Co-creation of responses to health crises with vulnerable communities, cultural sensitivity, self-determination, planetary health
Decolonising COVID-19	2020	The Lancet Global Health	UK	The Lancet Global Health	Journal	Commentary/Opinion piece	COVID-19, asymmetrical power structures within global health, racism and global health practice, the colonisation of medicine, economics, and politics
Decolonising Global (Public) Health: from Western universalism to Global pluriversalities	2020	Clara Affun-Adegbulu, Opemiposi Adegbulu	Belgium, UK	BMJ Global Health	Journal	Commentary/Opinion piece	Definition and conceptualisations of decolonising global health, Eurocentric conception of global health, decolonisation at epistemic and ontological levels
Decolonising global health	2024	Julia Ngozi Chukwuma	UK	The Companion to Development Studies (4th Edition)	Book	Commentary/opinion piece	COVID-19 highlighting global health inequities. Understanding colonial roots of global health informs practice today. Issues with neo-liberal capitalism and technical (rather than system) focus approach to 'solving' global health issues and role in reinforcing power imbalances.
Decolonising global health by decolonising academic publishing	2022	Shahzad Amjad Khan	Pakistan	BMJ Global Health	Journal	Commentary/Opinion piece	Academic publishing as a place to start decolonisation, hierarchies of evidence, need for transparency
Decolonising global health evaluation: Synthesis from a scoping review	2022	Ichhya Pant, Sonal Khosla, Jasmine Tenpa Lama, Vidhya Shanker, Mohammed AlKhaldi, Aisha El-Basuoni, Beth Michel, Khalil Bitar, Ifeanyi McWilliams Nsofor	USA, Canada, UAE, Switzerland, Palestine, Nigeria	PLOS Global Public Health	Journal	Scoping review	Defining decolonising global health evaluation, identifying themes and factors associated with decolonising global health evaluation, decolonising global health as a 'journey'
Decolonising global health in 2021: a roadmap to move from rhetoric to reform	2021	Mishal Khan, Seye Abimbola, Tammam Aloudat, Emanuele Capobianco, Sarah Hawkes, Afifah Rahman-Shepherd	UK, Australia, Switzerland	BMJ Global Health	Journal	Commentary/Opinion piece	Defining decolonising global, systemic or organisational change of global health institutions, steps required to decolonise, metrics for accountability
Decolonising global health in the Global South by the Global South: Turning the lens inward	2023	Dhananjaya Sharma, Nadia Adjoa Sam-Agudu	India, Nigeria, Ghana, USA	BMJ Global Health	Journal	Commentary/opinion piece	Global health actors from the global south and their role in decolonisation (from the perspective of authors from the global south), the importance of introspection regarding colonialism/decolonising, systemic inequalities, the structural nature of coloniality, individual behaviour, knowledge creation, frameworks for taking action on decolonising global health.
Decolonising global health in the time of COVID-19	2020	Mariam O Fofana	USA	Global Public Health	Journal	Literature review	Colonial inheritance of modern global health, COVID-19, steps for dismantling colonialism in global health
Decolonising global health research: Shifting power for transformative change	2024	Ramya Kumar, Rajat Khosla, David McCoy	Malaysia, Sri Lanka	PLOS Global Health	Journal	Commentary/opinion piece	Presentation of framework for understanding colonialism in global health research. Importance of equitable research partnerships. Call for structural change within global health research system to decolonise. Importance of transparent global health funding.
Decolonising global health: a Philippine perspective	2022	Edward Christopher Dee, Gideon Lasco	USA, Philippines	The Lancet	Journal	Commentary/Opinion piece	Rebuttal of criticism of decolonising global health, epistemic injustice, steps to decolonise, community empowerment
Decolonising global health: beyond ‘reformative’ roadmaps and towards decolonial thought	2021	Monica Mitra Chaudhuri, Laura Mkumba, Yadurshini Raveendran, Robert D Smith	Canada, USA, Switzerland	BMJ Global Health	Journal	Commentary/Opinion piece	Definitions of decolonising global health, colonialism within global health industry, need for comprehensive structural reform, necessity of reflection/introspection
Decolonising Global Health: Clarifying Concepts for Equitable Practice	2024	Emma Sophie Spanaus, Luis Eugenio De Souza	Switzerland, Germany, Brazil	Epidemiology & Public Health	Journal	Qualitative study/Literature Review	Power dynamics, importance of traditional and community-based knowledge, structural determinants of colonialism in global health, need for equitable partnerships, reform of global health curriculums
Decolonising global health: if not now, when?	2020	Ali Murad Büyüm, Cordelia Kenney, Andrea Koris, Laura Mkumba, Yadurshini Raveendran	USA	BMJ Global Health	Journal	Commentary/Opinion piece	Colonial structure of global/public health, individual duty, actions to decolonise global health, COVID-19, reflective practice, paradigm/leadership/knowledge shift
Decolonising global health: transnational research partnerships under the spotlight	2020	David S Lawrence, Lioba A Hirsch	UK, Botswana	International health	Journal	Literature review	Clinical trials, transnational research partnerships, decolonising global health research
Decolonising global health: where are the Southern voices?	2021	Samuel Oti, Jabulani Ncayiyana	Kenya, South Africa	BMJ Global Health	Journal	Commentary/Opinion piece	Definitions of decolonising global health, need for pragmatism over further theorising, decolonising from perspective of Global South
Decolonising global health: why the new Pandemic Agreement should have included the principle of subsidiarity	2024	Thana C de Campos-Rudinsky, Sarah L Bosha, Daniel Wainstock, Sharifah Sekalala, Sridhar Venkatapuram, Caesar Alimsinya Atuire	Chile, USA, Brazil, UK, South Africa, Ghana	The Lancet Global Health	Journal	Commentary/opinion piece	Pandemic Agreement, subsidiarity, recommendations for global health governance, engaging local and community level actors in global health policy and mechanisms
Decolonising ideas of healing in medical education	2020	Amali U. Lokugamage, Tharanika Ahillan, S. D. C. Pathberiya	UK	Journal of Medical Ethics	Journal	Literature review	Medical education, enlightenment, healing, indigenous knowledge, colonial history of global health
Decolonising integrative health: learning more from and elevating the voices of rich but often neglected health traditions	2022	Jon Wardle	Australia	Advances in Integrative Medicine	Journal	Commentary/Opinion piece	Decolonising integrative health and traditional medicine
Decolonising 'man', resituating pandemic: an intervention in the pathogenesis of colonial capitalism	2021	Rosemary J Jolly	USA	Medical Humanities	Journal	Commentary/Opinion piece	Decolonial pandemic history, individual and institutional practice for decolonisation
Decolonising public and planetary health, or Chthulecene mediations	2023	Jan Gresil Kahambing	China	Journal of Public Health	Journal	Commentary/Opinion piece	Structural inequalities in global health, funding, power asymmetries between actors from high and low income countries working in global health.
Decolonising qualitative research to explore the experiences of Manitoba's urban Indigenous population living with type 2 diabetes mellitus, obesity and bariatric surgery	2020	Krista Hardy, Kathleen Clouston, Marta Zmudzinski, Melinda Fowler-Woods, Geraldine Shingoose, Amanda Fowler-Woods, Felicia Daeninck, Andrew Hatala, Ashley Vergis	Canada	BMJ Open	Journal	Qualitative study	Decolonising qualitative research in practice, cultural and community engagement
Decolonising violence against women research: a study design for co-developing violence prevention interventions with communities in low and middle income countries (LMICS)	2021	Jenevieve Mannell, Safua Akeli Amaama, Ramona Boodoosingh, Laura Brown, Maria Calderon, Esther Cowley-Malcolm, Hattie Lowe, Angélica Motta, Geordan Shannon, Helen Tanielu, Carla Cortez Vergara	UK, New Zealand, Samoa, Peru	BMC Public Health	Journal	Case study	Decolonising violence against women research, power dynamics in western research practice, community participation and co-design
Decolonisation and antiracism: intersecting pathways to global health equity	2024	Collins O. Airhihenbuwa, Chandra Ford, Juliet Iwelunmor, Derek M. Griffith, Khadijah Ameen, Teri Murray, Ucheoma Nwaozuru	USA	Ethnicity & Health	Journal	Commentary/opinion piece	Decolonisation and antiracism, colonisation, health equity, public health critical race praxis, global structural racism
Decolonisation in health professions education: reflections on teaching through a transgressive pedagogy	2016	Ruth Rodney	Canada	Canadian Medical Education Journal	Journal	Qualitative study	Transgression in medical education that can lead to decolonising, acknowledging the HIC educators' positionality in LMICs, reflective practice, the historical colonial roots of global health
Decolonizing Epidemiological Research: A Critical Perspective	2023	Yusuff Adebayo Adebisi	UK	Avicenna Journal of Medicine	Journal	Commentary/opinion piece	Important role for indigenous and marginalized populations, power imbalances in epidemiological research, distribution of research funding, authorship in epidemiological research, journal editorship.
Decolonizing global health — what does it mean for us?	2023	Birger C. Forsberg, Jesper Sundewall	Sweden, South Africa	European Journal of Public Health	Journal	Commentary/Opinion piece	Mapping flow of funds in global health, research on use and efficiency of funding in global health, twinning of research programmes for HIC and LMIC based institutions.
Decolonizing global health by engineering equitable relationships	2024	Nature Reviews Bioengineering	Unspecified	Nature Reviews Bioengineering	Journal	Commentary/opinion piece	Bioengineering; equitable relationships to drive innovation; hierarchical assumptions and disregard/lack of understanding of local knowledge and contexts.
Decolonizing global health curriculum: from fad to foundation	2023	Anna Kalbarczyk, Sylvie Perkins, Sabreena N. Robinson, Mahnoor K. Ahmed	USA	Frontiers in Education	Journal	Qualitative study	Interpretation of decolonising global health in educational programmes, guidance, protected time and support for decolonising activities,
Decolonizing global health education: rethinking institutional partnerships and approaches	2021	Quentin G Eichbaum, Lisa V Adams, Jessica Evert, Ming-Jung Ho, Innocent A Semali, Susan C van Schalkwyk	USA, Tanzania, South Africa	Academic Medicine: Journal of the Association of American Medical Colleges	Journal	Commentary/Opinion piece	Intersectionality of colonialism, power dynamics and neo-colonialist assumptions, approaches to challenge colonial paradigms in global health
Decolonizing global health from the perspectives of global health actors in Low-middle Income Countries	2021	Sedem Adiabu	USA	Emory University Theses and Dissertations	Grey literature	Qualitative study	Interpretations of decolonising global health, political manipulation, hypocrite, distrust, partnership working, steps needed to decolonise
Decolonizing Global Health Research: Perspectives from US and International Global Health Trainees	2023	Matthew DeCamp, Limbanazo Matandika, Lameck Chinula, Jorge L Cañari-Casaño, C Hunter Davis, Emily Anderson, Marlena McClellan, Benjamin H Chi, Valerie A Paz-Soldan	USA, Malawi, Peru	Annals of Global Health	Journal	Mixed methods study	Exploration of research fellow's perceptions of decolonising, definitions, awareness and attitudes to decolonising global health,
Decolonizing Global Health: A critical perspective from Latin America	2024	Vivian Laurens, César Abadía-Barrero	USA	The Routledge Handbook of Anthropology and Global Health	Book	Commentary/opinion piece	Decolonising global health from the perspective of Latin American Social Medicine, disconnect between global health funders/leaders and local knowledge and understanding of needs. Neo-liberalisation of global health, emphasis on individual agency. Indigenous concept and practice of 'Buen Vivir' in rural communities in Colombia as an example of decolonial approach in action.
Decolonizing Global Health: A Moment to Reflect on A Movement	2021	Madhukar Pai	Canada	Forbes	Grey literature	Commentary/Opinion piece	COVID-19, the plurality of decolonising global health, need for structural change, awareness of decolonising global health in the Global South, need to understand the history and meaning of decolonising global health
Decolonizing Global Health: Increasing Capacity of Community Health Worker Programs	2023	Pamela Avila	USA	Annals of Global Health	Journal	Commentary/opinion piece	Community health workers, the ethnicity of global health volunteer experiences and researchers, sustainability
Decolonizing global health: what should be the target of this movement and where does it lead us?	2022	Xiaoxiao Kwete, Kun Tang, Lucy Chen, Ran Ren, Qi Chen, Zhenru Wu, Yi Cai, Hao Li	China	Global Health Research and Policy	Journal	Commentary/Opinion piece	Historical colonial context and its ongoing influence in global health, meaning of decolonising global health, systemic change, the development of decolonial practice
Decolonizing healthcare innovation: Low cost solutions from low-income countries	2024	Matthew Harris	UK	Decolonizing healthcare innovation: Low cost solutions from low-income countries	Book	Commentary/Opinion piece	Global health, innovation, decolonisation through the promotion of knowledge, expertise and innovation from contexts that have previously been neglected, HICs learning from and making use of innovation from LMICs.
Decolonizing Indigenous health: Generating a productive dialogue to eliminate Rheumatic Heart Disease in Australia	2021	Emma Haynes, Roz Walker, Alice G. Mitchell, Judy Katzenellenbogen, Heather D'Antoine, Dawn Bessarab	Australia	Social Science and Medicine	Journal	Qualitative study	Importance of cultural safety and reflexivity on power and privilege, systemic change, combining indigenous knowledge with biomedical and social science approaches
Decolonizing the decolonisation movement in global health: A perspective from global surgery	2022	Emmanuel Bua, Saad Liaqat Sahi	Uganda, USA	Frontiers in Education	Journal	Commentary/opinion piece	Discussion on decolonising global health by an author from a low and middle income country. Decolonisation of global surgery, barriers to health education in global surgery, issues with surgical missions to LMICs, knowledge sharing and bilateral exchange
Developing an agenda for the decolonisation of global health	2024	David McCoy, Anuj Kapilashrami, Ramya Kumar, Emma Rhule, Rajat Khosla	Malaysia, England, Sri Lanka	Bulletin of the World Health Organization	Journal	Commentary/opinion piece	Structural nature of power inequities within global health, importance of finance, the commodification of healthcare (demonstrated by profits made through COVID-19 response), social medicine as anticolonial tradition in Latin America, need for stronger role for WHO, more inclusive and diverse global health governance.
Dialogical reflexivity towards collective action to transform global health	2021	Harvy Joy Liwanag, Emma Rhule	Malaysia	BMJ Global Health	Journal	Commentary/Opinion piece	Reflexivity and what reflexivity requires in order to contribute to decolonising global health, building cultures of reflexivity, actions to decolonise global health
Diversifying Implementation Science: A Global Perspective	2022	Sophia M. Bartels, Shabab Haider, Caitlin R. Williams, Yameen Mazumder, Latifat Ibisomi, Olakunle Alonge, Sally Theobald, Till Bärnighausen, Juanita Vasquez Escallon, Mahnaz Vahedi, Rohit Ramaswamy, Malabika Sarker	USA, South Africa, Nigeria, UK, Germany, Switzerland, Bangladesh	Global Health: Science and Practice	Journal	Commentary/Opinion piece	Role of leadership in increasing the influence of researchers and practitioners from LMICs, equity in implementation research, community participation, global action
Do we need to decolonise global health?	2021	Tammam Aloudat	Switzerland	Action to Decolonise Global Health	Grey literature	Commentary/Opinion piece	Colonial legacies in global health, inequitable power structures, disparities in health outcomes, action to decolonise global health
Efforts to a Belief and Decolonise Global Health	2023	Salim Omambia Matagi	Kenya	Journal of Advances in Medicine and Medical Research	Journal	Qualitative study/Literature Review	Decolonisation of academic publishing and research, global health as a colonial tool, individual duty of global health actors to drive move away from colonial model of global health.
Eliminating the White Supremacy Mindset from Global Health Education	2022	Agnes Binagwaho, Brianna Ngarambe, Kedest Mathewos	Rwanda	Annals of Global Health	Journal	Commentary/Opinion piece	Roots of colonisation, white supremacy, acknowledgement of expertise within LMICs, epistemic injustice
Epistemic injustice in academic global health	2021	Himani Bhakuni, Seye Abimbola	Netherlands, USA	The Lancet Global health	Journal	Commentary/Opinion piece	Epistemic injustice in global health, research, academia
Forming a coalition of the willing to decolonise global health. Is it possible, what impact could it have, and what next?	2021	Development Reimagined	China, Kenya	Development Reimagined	Grey literature	Commentary/Opinion piece	Practical steps/a framework for decolonising global health, inequitable power dynamics, institutional scepticism about decolonising global health, lessons from COVID-19
Global Health and Decolonisation in Higher Education: Examining the Attitudes, Perceptions and Possibilities of Educators	2023	Amani Eltayb, Karin Båge, Abdalla Mohamed Ibrahim, Natalie Jellinek, Raman Preet, Zoe Säflund, Jennifer Valcke	Sweden, Somalia	Philosophy and Theory in Higher Education	Journal	Mixed methods study	Exploring perceptions and meaning of decolonisation amongst global health educators, value and relevance of decolonisation, decolonising and the relationship with global power structures.
Global health and the elite capture of decolonisation: On reformism and the possibilities of alternate paths	2023	Daniel W. Krugman	USA	PLOS Global Public Health	Journal	Literature review	Radical decolonial theory, social change, the "buzzwordification" of decolonising global health, the elite capture of decolonisation.
Global health education in high-income countries: confronting coloniality and power asymmetry	2022	Hoda Sayegh, Christina Harden, Hijab Khan, Madhukar Pai, Quentin G Eichbaum, Charles Ibingira, Gelila Goba	USA, Canada, Uganda	BMJ Global Health	Journal	Commentary/Opinion piece	Global health education, the changes needed to address power asymmetries in global health education
Global Health Partnerships and the Brocher Declaration: Principles for Ethical Short-Term Engagements in Global Health	2022	Shailendra Prasad, Myron Aldrink, Bruce Compton, Judy Lasker, Peter Donkor, David Weakliam, Virginia Rowthorn, Efua Mantey, Keith Martin, Francis Omaswa, Habib Benzian, Erwin Clagua-Guerra, Emilly Maractho, Kwame Agyire-Tettey, Nigel Crisp, Ramaswami Balasubramaniam	USA, Ghana, Ireland, Guatemala, Uganda, UK, India	Annals of Global Health	Journal	Commentary/Opinion piece	Short terms experiences in global health (HIC to LMIC) as a symptom of a colonising mindset in global health, COVID as a break and chance to recalibrate, individual duty in enacting change
Global Health: Reimagining Perspectives	2020	Fernando De Maio, Jonatan Konfino	USA, Argentina	Health Disparities in Allergic Diseases	Book	Commentary/Opinion piece	Human rights, epidemiology, collaboration and partnership working
Global mental health research and practice: a decolonial approach	2022	Eliut Rivera-Segarra, Franco Mascayano, Lubna Alnasser, Els van der Ven, Gonzalo Martínez-Alés, Sol Durand-Arias, Maria Francesca Moro, Elie Karam, Ruthmarie Hernández-Torres, Sebastián Alarcón, Alíxida Ramos-Pibernus, Rubén Alvarado, Ezra Susser	Puerto Rico, USA, Saudi Arabia, Netherlands, Spain, Mexico, Italy, Lebanon, Chile	The Lancet Psychiatry	Journal	Commentary/Opinion piece	The reproduction of power imbalances and patterns of oppression through global mental health, the need for individual action and active resistance, access to conversations on decolonising, epistemic justice, pragmatic solidarity and sovereign acts as routes to decolonising global mental health
Has Authorship in the Decolonizing Global Health Movement Been Colonized?	2023	Chris A. Rees, Gouri Rajesh, Hussein K. Manji, Catherine Shari, Rodrick Kisenge, Elizabeth M. Keating, Ikechukwu U Ogbuanu, Kitiezo Aggrey Igunza, Richard Omore, Karim P. Manji	USA, Tanzania, Sierra Leone, Kenya	Annals of Global Health	Journal	Qualitative study/Literature Review	Colonisation of authorship, understandings of decolonising in high and low and middle income countries, the need for representation from authors from low- and middle-income countries.
How and Why Should We Decolonise Global Health Education and Research?	2022	Ipek Demir	UK	Annals of Global Health	Journal	Commentary/Opinion piece	Health education, research, racism, the potential for decolonised global health curricula to be more rigorous and deliver better health outcomes, legacies of coloniality in health outcomes, actions for decolonising global health education
In the wake: Interpreting care and global health through Black geographies	2019	Lioba A Hirsch	UK	Area	Journal	Commentary/Opinion piece	Critical theoretical reflections on global health interventions in postcolonial societies
Is decolonisation sufficient?	2022	Sana Qais Contractor, Jashodhara Dasgupta	Belgium, Norway, India	BMJ Global Health	Journal	Commentary/opinion piece	Local and global power hierarches, intersectionality, the types of actors within global health, positionality.
Is it possible to decolonise global health institutions?	2021	Lioba A Hirsch	UK	The Art of Medicine	Journal	Commentary/Opinion piece	Structural determinants of power, structural violence and hurt inflicted by global health institutions, need for action rather than tokenistic efforts to decolonise
It's Time to Decolonise the Decolonisation Movement	2021	Ijeoma Nnodim Opara	USA	PLOS Blogs: Speaking of Medicine and Health	Grey literature	Commentary/Opinion piece	Power asymmetries, critical analyses of global health, who is currently benefitting from decolonising movement, steps needed to decolonise global health
Knowledge is Power: Assessing Academic Decolonisation through Bidirectionality of Training in Global Health Fellowships	2022	Rebecca Fujimara, Yalda Jabbarpour	USA	McGill Journal of Global Health	Journal	Literature review	The idea of 'bi-directional power', power imbalances between global north and south, the benefits of global health fellowships for those from high income countries.
Letter to the Editor: Leveraging ChatGPT to democratize and decolonise global surgery: Large language models for small healthcare budgets	2024	Hinpetch Daungsupawong, Viroj Wiwanitkit	Laos, India, Nigeria	World Journal of Surgery	Journal	Commentary/opinion piece	
Missing in action: a scoping review of gender as the overlooked component in decolonial discourses	2024	Tiffany Nassiri-Ansari, Emma Louise Margaret Rhule	Malaysia	BMJ Global Health	Journal	Qualitative study/Literature Review	Gender as an overlooked dimension within decolonising global health literature, intersectionality, colonial impact on concepts of identity and knowledge, the need for the integration of a gender lens within work to decolonise global health.
Offline: The case for global health	2023	Richard Horton	UK	The Lancet	Journal	Commentary/Opinion piece	Origins of global health, link between funding and influence, scientific publishing and access to journals, structural racism.
Offline: The myth of "decolonising global health"	2021	Richard Horton	UK	The Lancet	Journal	Commentary/Opinion piece	Neo-colonialism, the structural determinants of colonialism in global health, decolonising global health as a challenge, the need for action over rhetoric
One Health: A social science discussion of a global agenda	2022	Jean Estebanez, Pascal Boireau	France	Parasite	Journal	Commentary/Opinion piece	One Health and its roots in the colonial period, re-legitimisation of local and non-human/animal knowledge, decolonisation of One Health to prevent epidemic emergence,
Post-decolonisation: Global Health and Global Surgery's Coming of Age	2022	Bhavna Chawla, Judith Lindert, Dhananjaya Sharma,	Switzerland, Germany, India	The Indian Journal of Surgery	Journal	Commentary/Opinion piece	Global surgery, medical education, disconnect between global north and the 'beneficiaries' of global health, post-colonial mindset of those working in global health
Powerful ideas? Decolonisation and the future of global health	2022	Mark Hellowell, Patricia Nayna Schwerdtle	UK, Germany	BMJ Global Health	Journal	Commentary/Opinion piece	Knowledge, universalism, colonial origins of global health, asymmetries in the distribution of epistemic authority and decision making power in global health, structural determinants of power within global health, global health's Eurocentric conception of humanity, COVID-19 response
Prioritizing equity and inclusion in global health dermatology	2021	Aileen Y Chang, Miriam Laker-Oketta, Sarah J. Coates	USA, Uganda	International Journal of Women's Dermatology	Journal	Commentary/Opinion piece	Dermatology, medical education, actions to decolonise global health dermatology through research, training and service delivery, funding, epistemic injustice, representation, the burden practitioners and researchers from HICs place on the LMIC settings in which they may practice.
Radiologists' Role in Decolonizing Global Health	2024	Farouk Dako, Toma S. Omofoye, John Scheel	USA	Journal of the American College of Radiology	Journal	Commentary/opinion piece	Radiology and decolonising global health, capacity strengthening, inequitable access to technology, brain drain, parachute research.
Realist evaluation in times of decolonising global health	2022	Dimitri Renmans, Nandini Sarkar, Sara Van Belle, Clara Affun-Adegbulu, Bruno Marchal, Ferdinand C Mukumbang	Belgium, USA	The International Journal of Health Planning and Management	Journal	Commentary/Opinion piece	Role of realistic evaluation in decolonising global health, importance of engaging with power inequities in global health, overreliance on Western-based theories and knowledge, participatory research methods.
Reflections on ‘Decolonizing’ Big Data in Global Health	2022	Danya M Qato	USA	Annals of Global Health	Journal	Commentary/Opinion piece	The principles of decolonising global health should be applied to the collection and use of data in global health, given its important role in global health, COVID-19, leadership by people who 'populate the dataset', need for decolonising agenda to be clear and understandable, ownership of data
Reimagining global health: From decolonisation to indigenization	2021	Suzanne Hindmarch, Sean Hillier	Canada	Global Public Health	Journal	Commentary/Opinion piece	Decolonisation, indigenous ontologies and health expertise, dominance of western principles and ideologies in global health
Rethinking development interventions through the lens of decoloniality in sub-Saharan Africa: The case of global health	2022	L Gautier, Y Karambe, J-P Dossou, O M Samb	Canada, Mali, Benin, Belgium	Global Public Health	Journal	Commentary/Opinion piece	Western dominance of global health, approaches to decolonising global health, the inability of global health to engage with the 'structures and mental models' of African populations.
Shifting Power in Global Health - Decolonising discourses - Series Synthesis	2022	Tiffany Nassiri-Ansari, Emma Rhule	Malaysia	Summary of United National University, Development Reimagined, and Wilton Park Virtual Dialogue Series	Grey Literature	Commentary/opinion piece	Exploration of decoloniality through language, representation, and positionality, funding, power dynamics, agency and equity as tools to reimagine collective development.
Swampscott in International Context: Expanding Our Ecology of Knowledge	2016	Christopher C. Sonn	Australia	American Journal of Community Psychology	Journal	Commentary/Opinion piece	Colonisation of knowledge, the 'dynamics of dominance and marginality in knowledge production in psychology', opportunities to decolonise community psychology.
Talk the talk and walk the walk: a novel training for medical students to promote decoloniality in global health	2024	Leah Ratner, Shela Sridhar, Sheila Owusu, Samantha L. Rosman, Rose L. Molina, Jennifer Kasper	USA, Ghana	Frontiers in Education	Journal	Mixed methods study	Decoloniality within medical education, moving from theory to practical application of decolonial principles, need for accountability and collective action, need for institutional and structural as well as individual-level change.
Talking Points: Catherine Kyobutungi on global health	2022	Helga Groll	UK	eLife	Grey literature	Commentary/Opinion piece	Helicopter models of research, mismatch between the priorities of funders and those based in Africa, need to move from discussion to action to decolonise.
The ‘decolonisation of global health’ agenda in Africa: harnessing synergies with the continent's strategic aspirations	2023	Aloysius Ssennyonjo, Phillip Wanduru, Elizabeth Omoluabi, Peter Waiswa	Uganda, Belgium, Sweden, Senegal, South Africa	European Journal of Public Health	Journal	Commentary/opinion piece	Call for Africa-based development practitioners to 'decolonise from within', need for egalitarian partnership working, need for African-led, African based models for capacity strengthening.
The activists trying to 'decolonise' global health	2019	Andrew Green	Germany	DevEX	Grey literature	Commentary/Opinion piece	Activism, race and political economy in the delivery of health services, structural determinants of global health, decolonial theory
The cognitive dissonance discourse of evolving terminology from colonial medicine to global health and inaction towards equity - A Preventive Medicine Golden Jubilee Article	2022	Delivette Castor, Luisa N Borrell	USA	Preventive medicine	Journal	Qualitative study	Language, equity/inequity, social justice, decolonising global health terminology
The feudal structure of global health and its implications for decolonisation	2022	Vikash Ranjan Keshri, Soumyadeep Bhaumik	Australia, India	BMJ Global Health	Journal	Commentary/Opinion piece	Euro-American hegemony over knowledge ecosystems in global health is supported by global health structures similar to old feudal power structures from colonial era, the need to dismantle structure of global health to move decolonising global health forward, parachuting-in of skills from high income to low and middle income countries, the colonial historical roots of global and international health
The foreign gaze: authorship in academic global health	2019	Seye Abimbola	Australia	BMJ Global Health	Journal	Commentary/Opinion piece	Entrenched power asymmetries in global health partnerships, the 'foreign gaze' in global health and its consequences and influence.
The future of global health education: training for equity in global health	2016	Lisa V. Adams, Claire M. Wagner, Cameron T. Nutt, Agnes Binagwaho	USA, Switzerland, Rwanda	BMC Medical Education	Journal	Literature review	Need for partnership working between the global north and global south, traditional (colonial) roles institutions from the global north and south play, models for equitable training partnerships in global health
The Rhetoric of Decolonizing Global Health Fails to Address the Reality of Settler Colonialism: Gaza as a Case in Point	2024	Eivind Engebretsen, Mona Baker	Norway, UK	International Journal of Health Policy and Management	Journal	Commentary/Opinion piece	Decolonising knowledge translation, colonial structures of health knowledge system, need for action, reflection and theorisation, need for clear definitions of decolonising global health.
The rise of non-communicable disease (NCDs) in Mozambique: decolonising gender and global health	2021	Claire Somerville, Khatia Rebeca Munguambe	Mozambique, Switzerland	Gender & Development	Journal	Qualitative study	Non-communicable disease, gender, healthcare systems, Mozambique, dominance of global North in setting global health agenda, structural inequalities in global health, biomedical explanatory models of illness and their ‘fit’ with existing or local models of illness and diagnosis.
The truth about decolonising global health worth spreading	2022	Juliet Iwelunmor	USA	The Lancet	Journal	Commentary/Opinion piece	Decolonising global health will take a long time, the process of decolonising, struggle, decolonising as an 'unconventional movement'
The way forward in decolonising global health	2023	Sudeep Adhikari, Irene Torres, Elizabeth Oele	Nepal, Ecuador, Kenya	The Lancet Global Health	Journal	Commentary/opinion piece	Recommendations for decolonising global health; reduce migration of health workers from LMICs, globalise clinical trials.
The words we choose matter: recognising the importance of language in decolonising global health	2021	Franziska Hommes, Helena Brazal Monzó, Rashida Abbas Ferrand, Meggan Harris, Lioba A Hirsch, Emilie Koum Besson, John Manton, Toyin Togun, Robindra Basu Roy	UK, Germany, Zimbabwe, Spain, Gambia	The Lancet Global Health	Journal	Commentary/Opinion piece	Importance of language and the terminology used to discuss global health, publishing in English and difficulties with translation, Anglocentrism as a barrier for readers, authors and researchers, the historical context of domination, dependence, and subordination in global health
Theories, models and best practices for decolonizing global health through experiential learning	2023	Steven R. Hawkes, Jenna L. Hawks, Heather S. Sullivan	USA	Frontiers in Education	Journal	Literature review	Experiential and transformative learning, critical reflection, educational practice, collaboration with local populations and community partners.
Theory from the South: a decolonial approach to the psychology of global inequality	2017	Glenn Adams, Sara Estrada-Villalta	USA	Current Opinion in Psychology	Journal	Commentary/Opinion piece	Coloniality within global health and 'modernity and modern mentalities', global security
Thinking the post-colonial in medical education	2008	Alan Bleakley, Julie Brice, John Bligh	UK	Medical Education	Journal	Commentary/Opinion piece	Post-colonial theory and medical education, the western medical curriculum's influence globally and its influence on alternative or local approaches to medicine and medical education
To Decolonise Global Health, We Must Examine the Global Political Economy	2021	Pooja Yerramilli	USA, India	Think Global Health	Grey literature	Commentary/Opinion piece	COVID-19, international aid, the commercial determinants of health, public sector spending, the prioritisation of financial sustainability over access to health care
To decolonise global surgery and global health we must be radically intentional	2023	Denis A. Foretia	Cameroon	The American Journal of Surgery	Journal	Commentary/Opinion piece	Medical tourism, health system strengthening, power dynamics within clinical teams, saviourism.
Toward a decolonised healthcare ethics: Colonial legacies and the Siamese crocodile	2020	Luis Cordeiro-Rodrigues	China	Developing world bioethics	Journal	Commentary/Opinion piece	COVID-19, interdependency, colonial legacy of hostility, global distribution of resources to support healthcare
Towards attainment of Indigenous health through empowerment: resetting health systems, services and provider approaches	2021	Cheryl Barnabe	Canada	BMJ Global Health	Journal	Case study	Colonial policies and their implications for the health of indigenous populations of commonwealth countries, imposition of models of healthcare without regard to context or indigenous knowledge, community led healthcare delivery
Transcending the guilt of global health	2019	Richard Horton	UK	Lancet	Journal	Commentary/Opinion piece	Academic institutions and their colonial legacies, barriers to academic publication for those from low-income and middle-income countries, epistemic injustice, the power of journals and their editorial staff
Transformational learning to decolonise global health	2021	Amali U Lokugamage, Sarah H M Wong, Nathan M A Robinson, Sithira D C Pathberiya	UK	The Lancet	Journal	Commentary/Opinion piece	Colonial era power hierarchies in global health, prerequisites for systemic change, programmes of transformational learning to decolonise global health
Transforming global health through equity-driven funding	2021	Jacob O Olusanya, Olufunmilayo I Ubogu, Fidelis O Njokanma, Bolajoko O Olusanya	Nigeria, South Africa	Nature Medicine	Journal	Commentary/Opinion piece	The philosophy of global health reinforces inequalities and promotes the interests of the Global North, funding structures and flows do not reflect equality, equity, diversity and inclusion (EEDI) principles, the steps that funders can take to decolonise global health
Transforming global health: decoloniality and the human condition	2024	Raphael Lencucha	Canada	BMJ Global Health	Journal	Commentary/opinion piece	Risk of the 'totalising gaze' by focussing solely on decolonising without recognition of complex human behaviour e.g. tendency to seek power, emphasise difference. Structural and epistemic inequalities in global health.
Undoing supremacy in global health will require more than decolonisation	2020	Keerti Gedela	UK	The Lancet	Journal	Commentary/Opinion piece	Global health architecture shaped by supremacy and colonisation, LMIC internal power relations, personal interest and systemic change
Undoing supremacy in global health will require more than decolonisation - Authors' reply	2021	Seye Abimbola, Madhukar Pai	Australia, Canada	Lancet	Journal	Commentary/Opinion piece	Colonialism and power asymmetry between high-income countries and low-income and middle-income countries, lessons from COVID-19, research practice
Unequal ecosystems of global health authorial expertise: Decolonising noncommunicable disease	2021	Clare Herrick, Oritsematosan Okpako, James D.A. Millington	UK	Health & Place	Journal	Literature review	Authorship on papers written about non-communicable diseases, the geographies of authorial expertise and its reproduction of the inequities of coloniality.
We Need to Decolonise	2022	Barry, Michele	USA	Stanford Centre for Innovation in Global Health: Global Health Spotlight Newsletter	Grey literature	Commentary/Opinion piece	Legacy of imperial power, inequitable nature of partnerships in global health between Global North and South, structural inequities and links to poorer outcomes, need to dismantle imperial power structures, need for self-scrutiny and openness to change.
What Do Global Health Practitioners Think about Decolonizing Global Health?	2022	Madelon L Finkel, Marleen Temmermann, Fatima Suleman, Michele Barry, Melissa Salm, Agnes Bingawaho, Peter H Kilmarx	USA, Kenya, South Africa, Rwanda	Annals of Global Health	Journal	Literature review	Perceptions of equity and power imbalances in global health partnerships, leadership, funding, authorship and academic initiatives as target areas for decolonisation
What Is Global Health: Science and Practice Doing to Address Power Imbalances in Publishing?	2020	Sonia Abraham, Stephen Hodgins, Abdulmumin Saad, Madeleine Short Fabicc	USA, Canada	Global Health: Science and Practice	Journal	Commentary/Opinion piece	Decolonising academic publication, epistemic injustice, local of power structures located in high-income countries while its implementation is located in LMICs, colonial history, funding, and social and economic structures, asymmetries of power and expertise, the influence of COVID-19.
What research evidence can support the decolonisation of global health? Making space for deeper scholarship in global health journals	2023	Sudha Ramani, Eleanor Beth Whyle, Nancy Kagwanja	India, South Africa, Kenya	Lancet Global Health	Journal	Commentary/opinion piece	Decolonising research practice, the important of local context and the importance of including experiential and local knowledge in global health interventions, need to challenge power imbalances and hegemonic discourses, systemic change in global health, importance of moving beyond biomedical approach and primacy of clinical trials or observational studies to incorporate qualitative and interpretive methods.
Why ‘elevating country voice’ is not decolonizing global health: A frame analysis of in-depth interviews	2023	Michael Kunnuji, Yusra Ribhi Shawar, Rachel Neill, Malvikha Manoj, Jeremy Shiffman	Nigeria, USA,	PLOS Global Health	Journal	Qualitative study/Literature Review	Decolonising global health's focus on addressing legacy of colonialism, neoliberal exploitation, emphasis on systemic change. Feasibility and desirability of wholesale decolonisation of global health.
Why and for whom are we decolonising global health?	2021	Ong'era F Mogaka, Jenell Stewart, Elizabeth Bukusi	Kenya, USA	The Lancet Global Health	Journal	Commentary/Opinion piece	Donor programmes, shift to local ownership, need for radical transformation and systematic change, white supremacy and the vested interests of established global health practitioners in high income countries, structural determinants of inequities within global health
Why It's important for future healthcare professionals to understand 'decolonizing global health', and how this can be done	2020	Nardin Farag	Canada	McGill University Perspectives Blog	Grey literature	Commentary/Opinion piece	Conceptualisation and understanding of decolonising global health, structural determinants of power in global health, need for reflective practice in global health, the repercussions of colonial history within global health
Will global health survive its decolonisation?	2020	Seye Abimbola, Madhukar Pai	Australia, Canada	The Lancet	Journal	Commentary/Opinion piece	Colonial roots of global health, representation within global health leadership, equitable and just global health architecture and how that compares to global health structures currently in place.

**Fig. 1:**
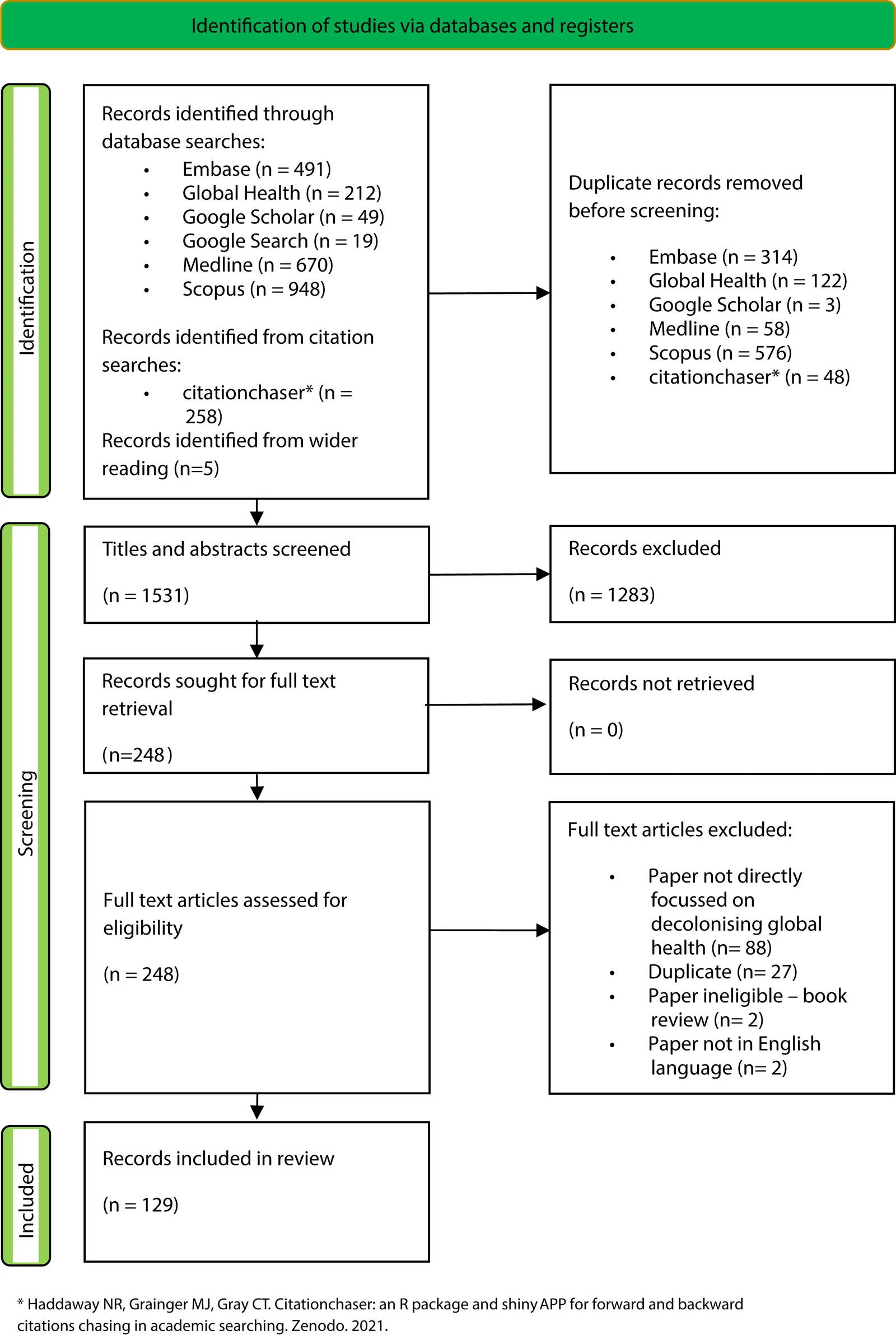
PRISMA Flow Diagram for the identification of studies and papers for inclusion in the review.

The majority of the papers included in our review were commentary or opinion pieces (n = 95). However, non-commentary and empirical literature comprised over a quarter of the evidence base (n = 34, 26.4%), including literature or scoping reviews (n = 13), qualitative studies (n = 15), mixed methods studies (n = 4) and case studies (n = 2). Authors of the included articles were affiliated with institutions from 56 different countries, with most authors (n = 53) affiliated to institutions in the USA, the UK (n = 30), and Canada (n = 17). Most papers included in our review were published in peer-reviewed journals (n = 112), with thirteen papers published in the non-peer-reviewed grey literature and four published in books. Articles included in the review were published between 2008 and 2024.

### 3.1 Who is talking about decolonising global health?

The impact of colonialism on health is far reaching, and as such, has attracted interest and commentary across many health-related sectors. These include psychology [30], dermatology [31], surgery [32], [33], [34], radiology [35], linguistics [36], public health [37], [38], [39], epidemiology [29], One Health [40], mental health [41], [42], bioengineering [43], rheumatic heart disease [3], health crises [44], healthcare innovation [45], academic medical centres [46], emergency medicine [47], [48] and global health institutions [49], [50]. Historical perspectives have been drawn upon [51] as have those regarding equity-driven funding [52] and global political economy [53].

Decolonising the research sector has attracted commentary, including research approaches to big data [54], [55], implementation science [56], realist evaluation [57] and perceptions of research fellows [58]. Similarly, research partnerships [18], [37], [59], [60] and publishing [61], [62], [63] have drawn critique.

Finally, many papers have focussed on decolonising the education sector, including global health learning [17], [34], [37], [50], [64], [65], [66], [67], [68], [69], [70], [71], [72], health care professional education [67], [73], [74] and educational partnerships [75], [76].

### 3.2 What is the geographical distribution of authors?

Despite the variety of sectors involved in decolonising global health, the location of authors (according to the primary institution that the author is affiliated with at the time of publication) shows a disparity in where the literature is being produced. Far more papers (n = 51, 40%) were produced by a single author based in a HIC than by a single author based in a LMIC (as per World Bank classifications) (n = 8, 6%). Articles with multiple author locations, produced in collaboration between HIC based authors and LMIC based authors made up 35% (n = 45) of the papers. Papers with multiple author locations exclusively from HICs made up 13% (n = 16), and papers with multiple author locations exclusively from LMICs made up 6% (n = 8). In HIC-LMIC research collaborations, it is also important to consider authorship hierarchies. In these collaborations, 69% (n = 47) had a first author based in a HIC; 22% (n = 15) had a first author based in an LMIC; and 7% (n = 5) had joint first authors based in a HIC and LMIC. Fig. 2, Fig. 3 set out a map showing the location of the authors and lead authors of included articles.

**Fig. 2:**
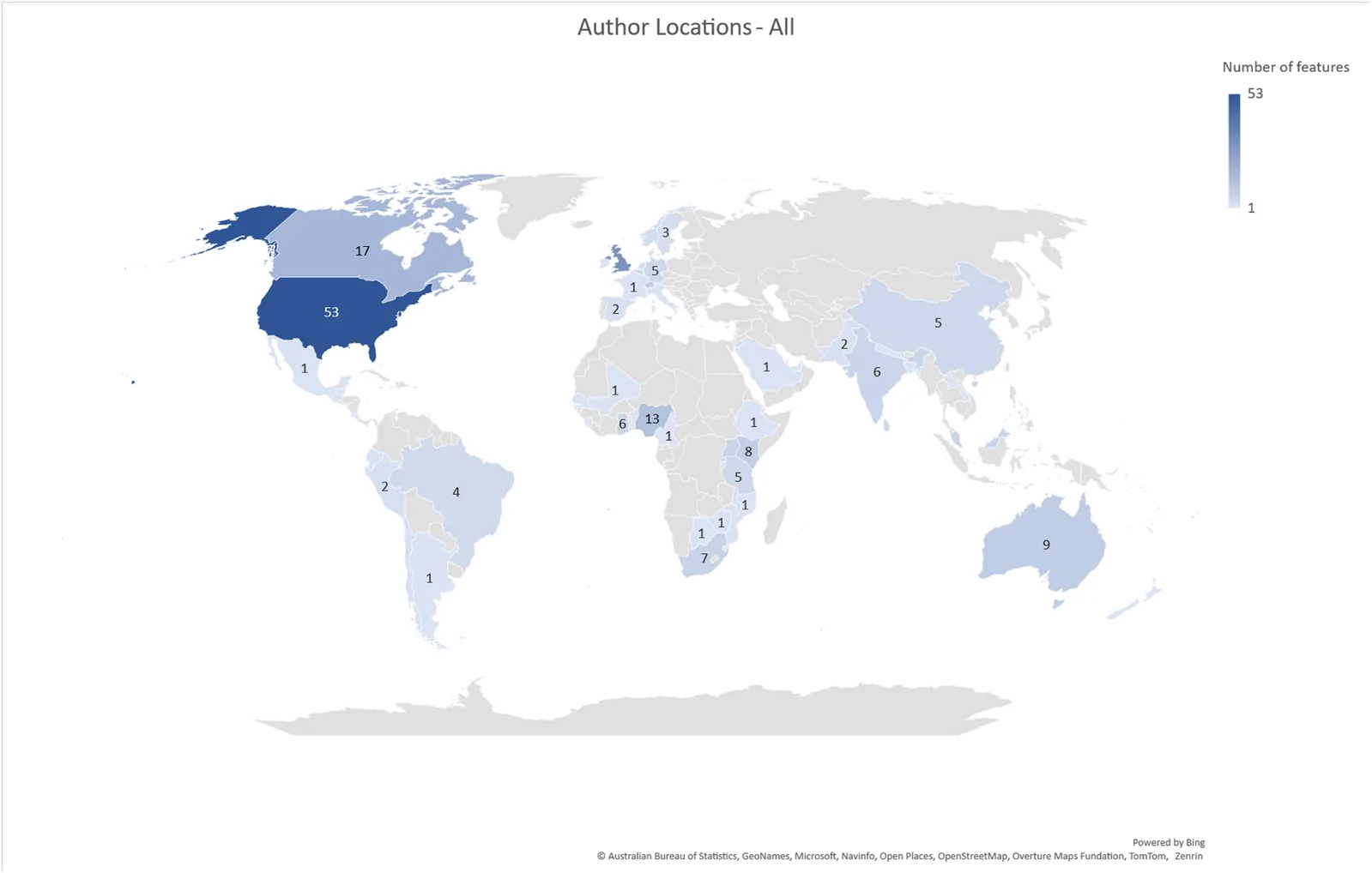

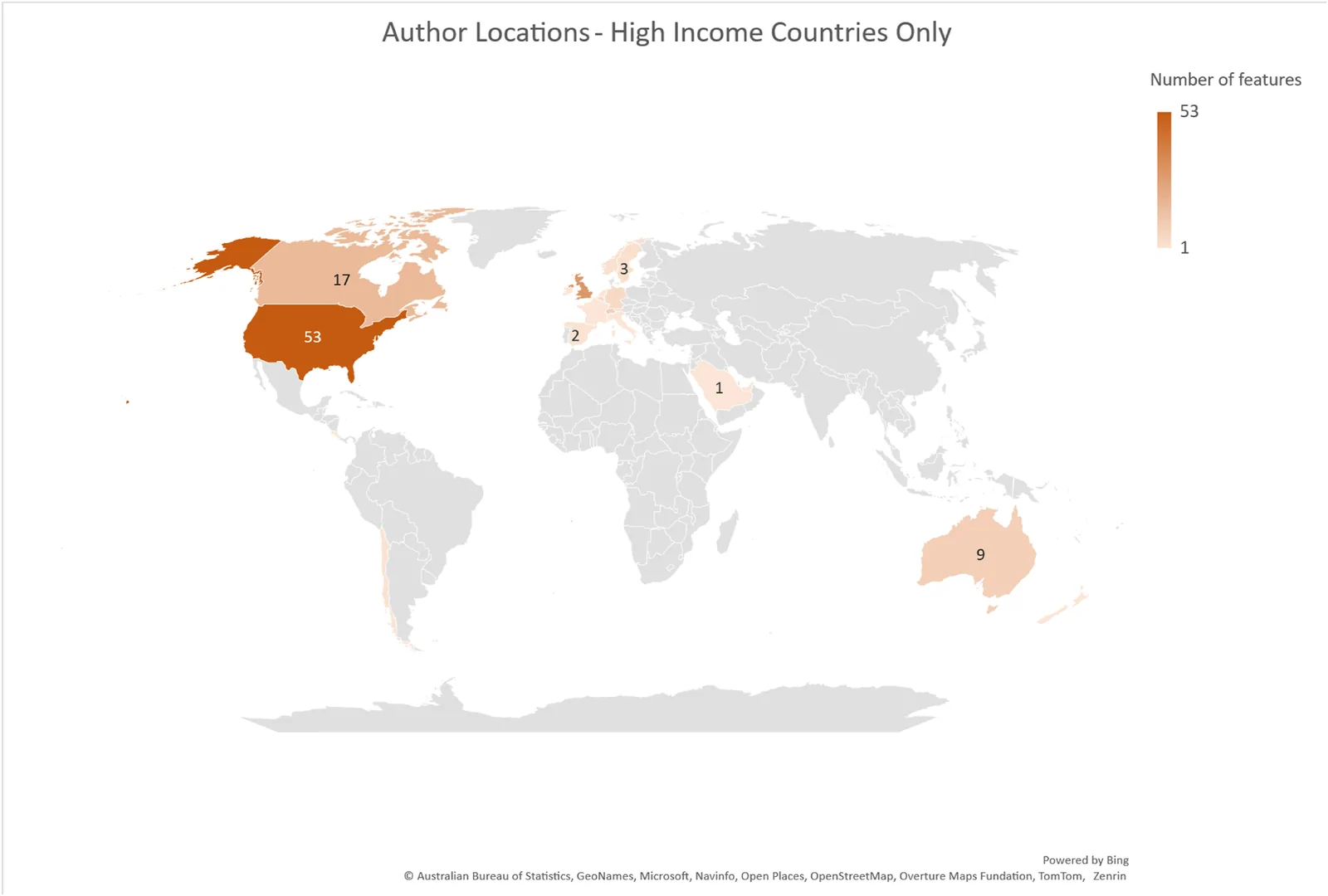

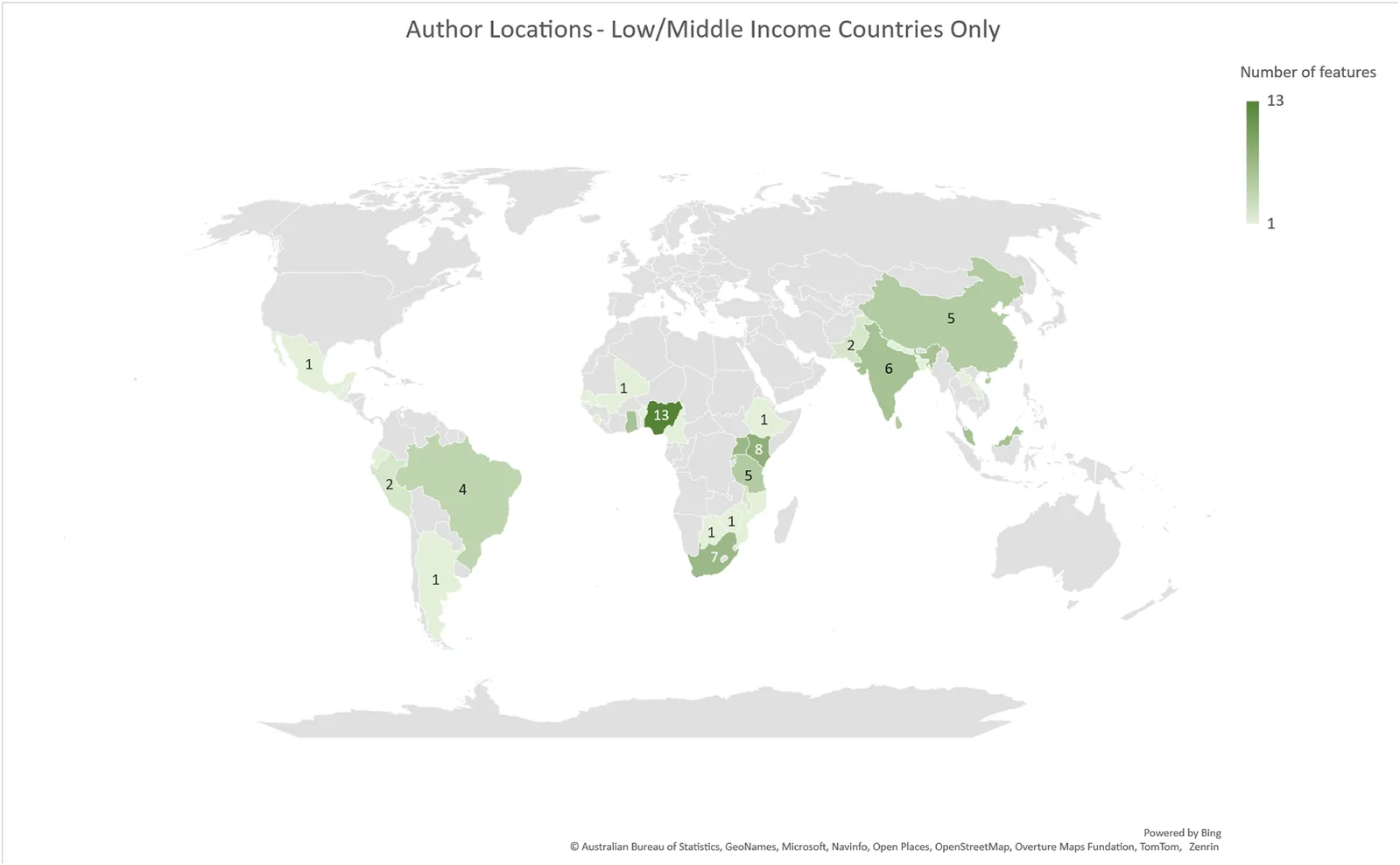
Location of the authors contributing to papers included in the review by World Bank income level* * Definition of author location refers to the primary institution that the author is affiliated with at the time of publication.

**Fig. 3:**
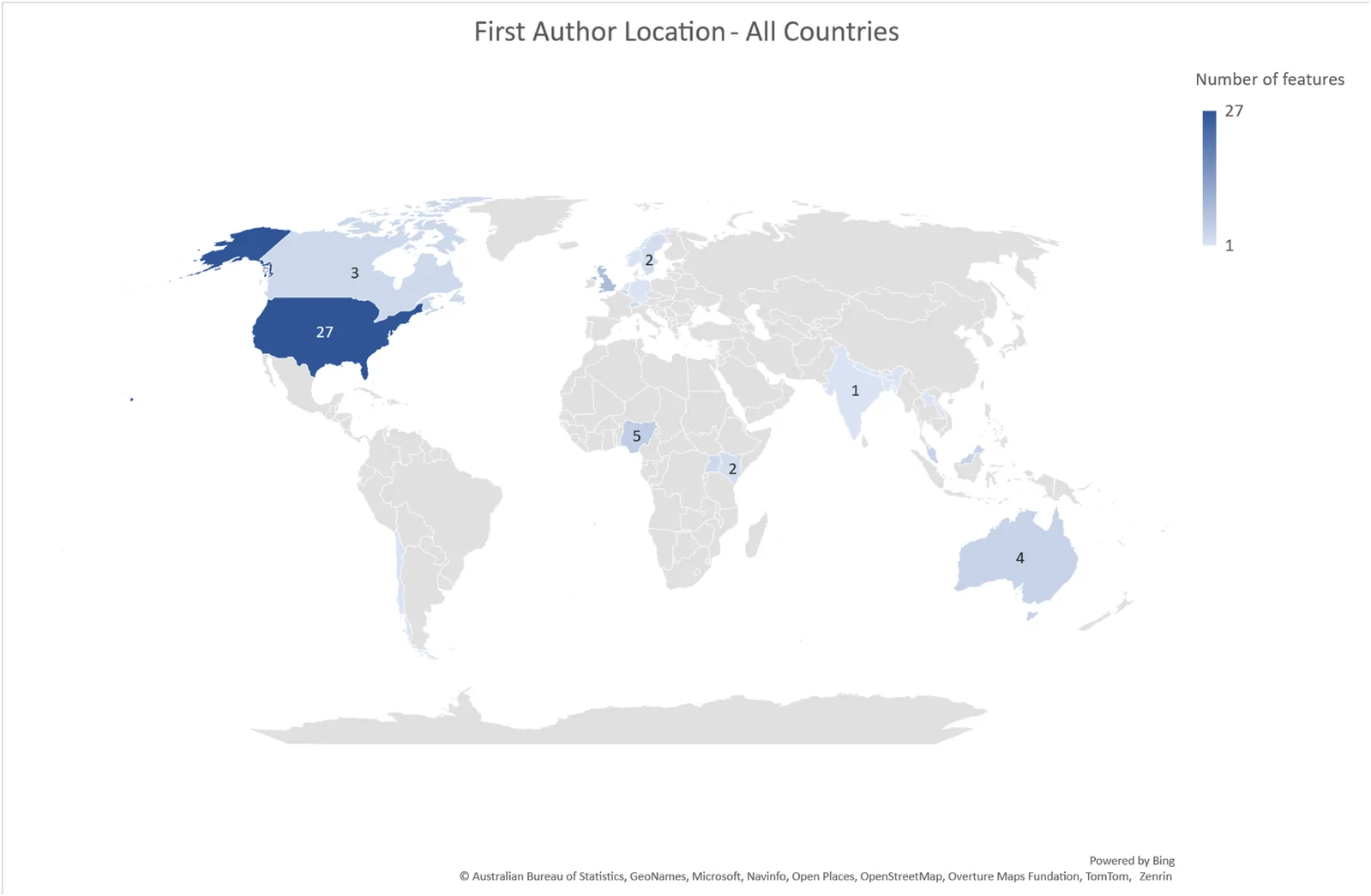

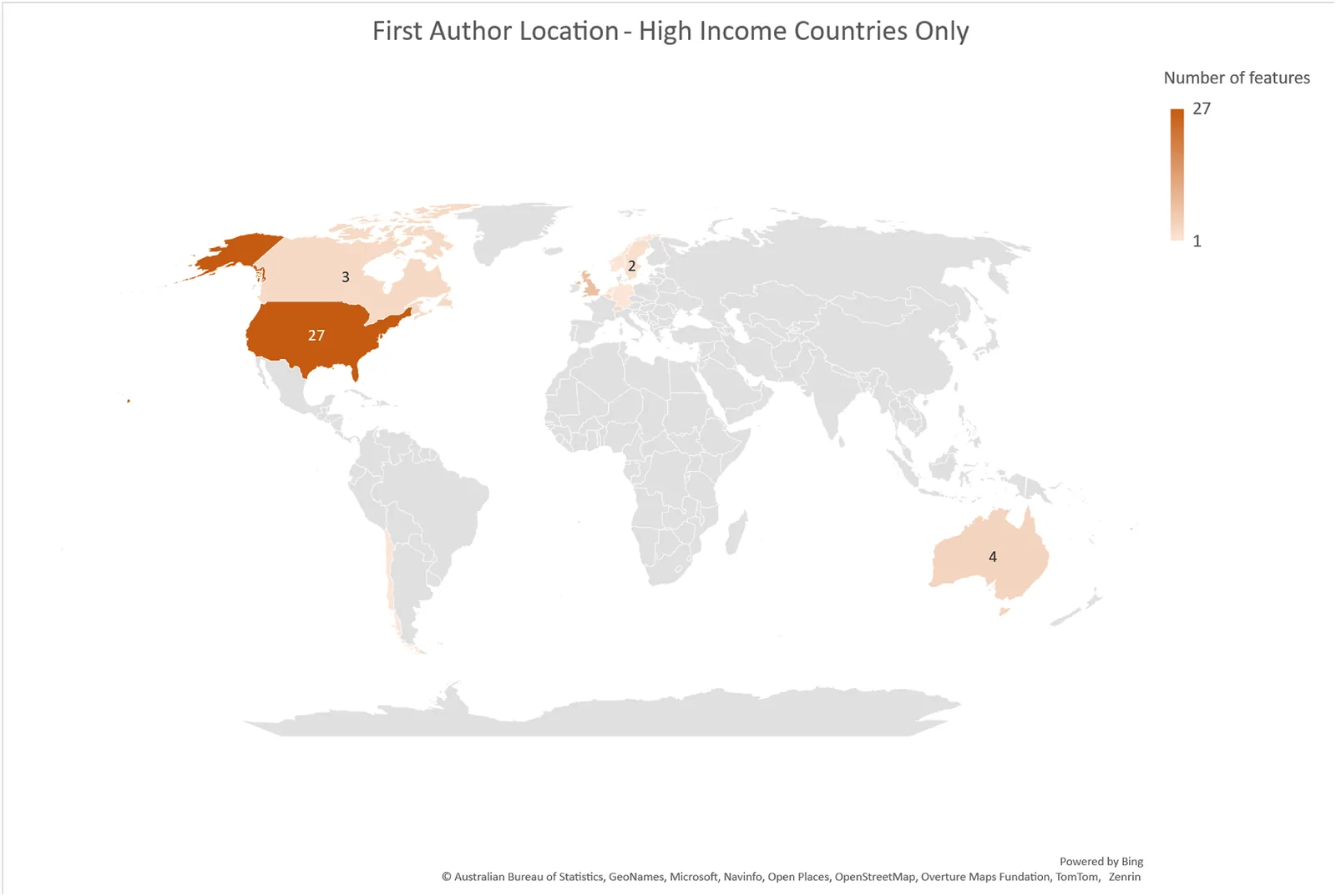

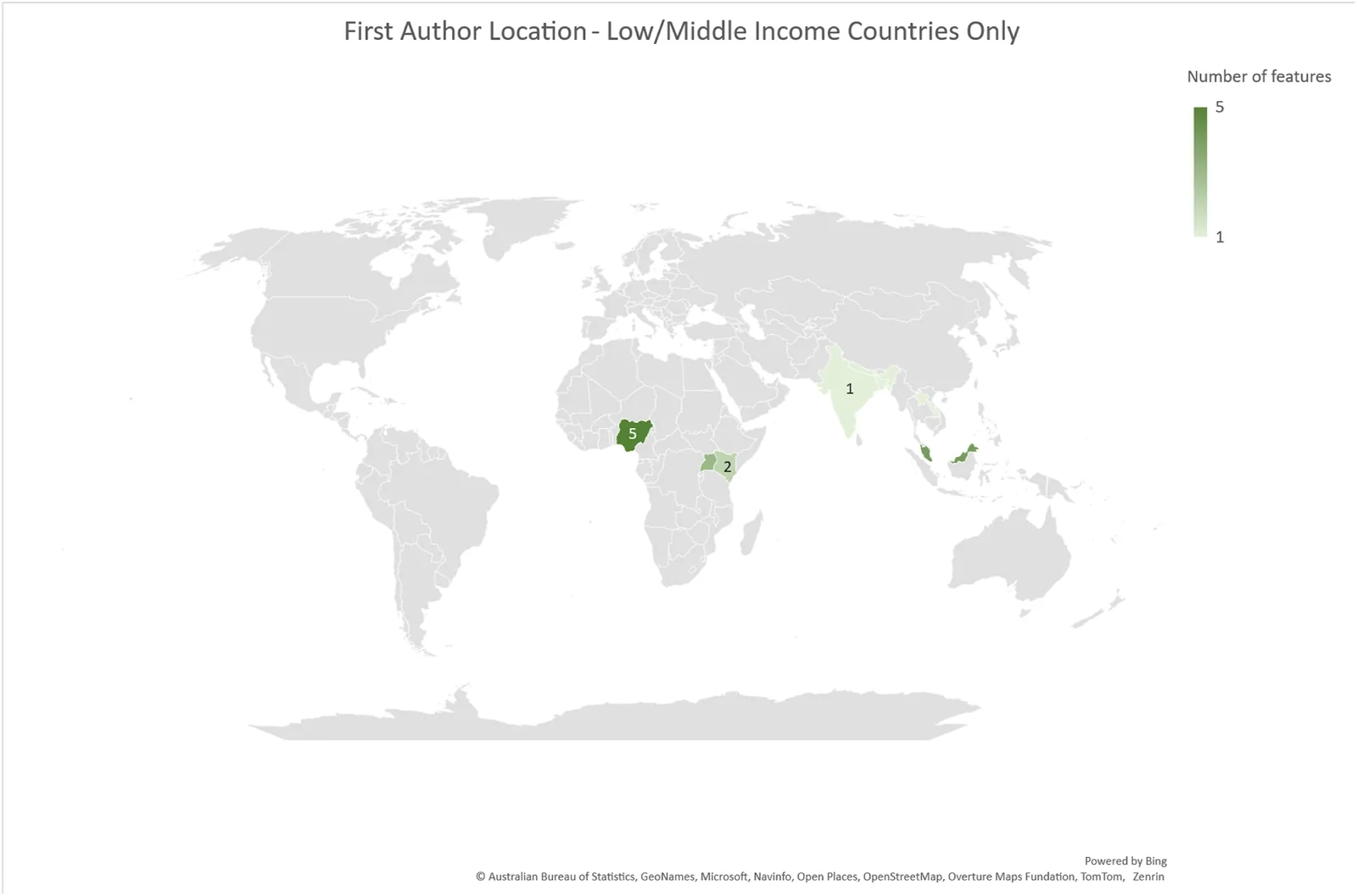
Country of origin for the first author of papers with multiple authors included in the review by World Bank income level **. ** This data only includes papers with more than one author. Note: Further details on the numbers of authors from each country are provided at the S2 Appendix.

### 3.3 Defining the decolonising global health movement

Decolonisation has been defined as the elimination of the colonial experience and its legacy [65] to allow for the independence and full agency of all involved organisations, communities, and persons [77]. When applied to global health, the decolonising movement seeks, amongst other things, to acknowledge global health's roots in colonialism [22]; highlight and challenge the power asymmetries in the global health architecture [7]; remove all forms of supremacy within spaces of global health practice [9]; identify ways in which global health teaching and research can overcome its colonial past [18], [49]; and advocate for critical global health education grounded in anti-colonial perspectives [7], [78].

The literature draws both clear distinctions and links between decolonising global health and other similar movements, frameworks and concepts, including the Black Lives Matter movement (n = 2) [7], [70], equity, diversity and inclusion (EDI) and anti-racist reform (n = 2) [49], [79]. Decolonising global health can be understood as an approach to social justice that also intersects with other harmful ‘-isms’, that perpetuate health inequities, such as racism, sexism, capitalism [16]. Unlike EDI initiatives, which often focus on representation and inclusion, decolonising global health advocates for addressing the root causes of structural and individual racism [8]. For example, the Editors of the Lancet Global Health pointed to the re-emergence of racist stereotypes towards Africans following the COVID-19 pandemic [5], demonstrating the intersectional nature of decolonising global health. Nassiri-Ansari and Rhule emphasise the intersection between race and gender and state that efforts to decolonise global health must focus on both race and gender equality [11]. However, the terminology of decolonising global health is far from universally accepted, lacks clarity [80], is poorly understood [72] and viewed as unconventional [81]. Krugman challenges the *"buzzwordification"* of decolonising global health, emphasising that decolonisation is a word with underdetermined and contested meanings, associations and representations [79], [82].

The complexity of what it means to decolonise global health should also be acknowledged. Contractor and Dasgupta use the example of India's caste system to draw attention to the local complexities of decolonisation, warning that the Global South is made up of diverse societies and a one-size-fits all approach is not appropriate. They draw attention to the historic cultural imbalances of power which were used in India by the colonisers and which will remain if decolonisation occurs without representation from suppressed minorities [83].

The terminology of decolonising global health has generated debate, not only around its definition but also regarding whether it should be used and what alternatives might be more appropriate. Critics argue that the term is predominantly defined by those from HICs, particularly within academic institutions, rather than by those at the receiving end of global health interventions. This, some authors suggest, perpetuates existing power and knowledge structures [7], [18], [49], [58]. For instance, Engebretsen critiques the decolonising global health movement, asserting that its rhetoric fails to address the root causes of pressing health crises, such as the disastrous situation in Gaza and the West Bank [84].

Hellowell [85] queries whether a decolonising global health framework is the best solution to issues within global health. A problem with this terminology is that it is associated with binary arguments which place people into groups of oppressed and oppressors depending on their background or place of origin. He suggests that solutions derived through this lens have the potential to harm the aims of the global health agenda by: ‘*(i) undermining confidence in scientific knowledge; ii) accentuating inter-group and inter-national antagonisms; and (iii) by discounting the degree of progress already achieved that may curtail opportunities for redistributive change in the future*’.

Binagwaho et al. provide an alternative to decolonising global health, arguing that the *“elimination of a white supremacy mindset”* is a better positioned term, as it recognises the crux of colonisation as assumed racial superiority, and how the legacy of such sustains the privilege white people enjoy at the expense of non-white people [65] regardless of colonial roots [86]. Whilst this review uses the decolonising global health terminology throughout, as this was reflected in the search terms and majority of the literature, further exploration of what other terminology is used is included in the discussion.

### 3.4 The history and legacy of colonial health

In terms of understanding the concept of decolonising global health, over 20% of manuscripts interrogated the origins [4], [9], [65], [87], [88], [89], [90], [91], [92], [93] and impact [9], [22], [37], [41], [49], [53], [65], [69], [87], [89], [90], [91], [92], [93], [94], [95], [96], [97] of colonial health; the key aspects are summarised here.

The discipline of colonial health developed from European colonialists in the 16th and 17th centuries, who saw health conditions unfamiliar to colonists as a threat to their mission. For instance, by the late 1800s, malaria was considered to be the largest obstacle to colonisation, with metropolitan military and business interests being compromised by the susceptibility of white settlers to malaria, which was by far the largest cause of death for that group [87]. In order for the colonial project to succeed, treatments for diseases impacting colonial administrators as well as the local populations whose labour they exploited were required [4].

In addition to developing strategies to protect the interests of colonisers, the necessity to understand ‘new’ disease aetiologies, patterns, and treatments of the indigenous populations, was usually pitted against the interests of the colonised [88]. Colonial authorities saw themselves as having the right solutions and rarely considered that the people they were trying to ‘help’ could possibly have their own solutions [65]. Most indigenous forms of understanding were devalued and demonised and were soon replaced by ‘technocratic experts’ who showed little concern for the socioeconomic realities on the ground [49], [89], [90]. Epistemic dominance from HICs carved its place. For example, the journal the Lancet was born as a product of colonialism and, at least in part, as an instrument to support and advance British imperial objectives [22].

The legacy of colonial health remains. The creation and dominance of colonial, missionary and tropical medicine followed by international health and now global health, continue to be underpinned by the remnants of colonial heritage and practices [4], [9], [49], [87], [91], [92], [93], [94]. These colonial practices have been *“codified into modernity, modern states, economic, social, intellectual and international institutions”* [69] and these institutions continue to reinforce racism and bias [37]. For instance, population refusal to seek care as a result of colonial violence, continues to hamper modern day medical emergencies [95], whilst educational curricula carry epistemological biases [69], including the exclusion of a ‘Southern’ perspective in intervention design and evaluation research approaches [96]. In a qualitative inquiry applying power analysis to global health project cycles, Tuhebwe and colleagues argue that colonial power dynamics can be seen throughout the project cycle of many global health programmes in terms of project design, funding allocation, and implementation [97].

By ignoring history, and continuing to accept power imbalances, patterns of oppression and exploitation reproduce and support the current system of global health [41]. This includes a distinct power imbalance where ‘resource limitations’ in low-income settings have always been externally imposed [53].

### 3.5 Why decolonise global health?

At the heart of why authors advocate that global health must be decolonised is the broad and consistent understanding that while colonisation is largely a remnant of the past [75], [98], the heritage and legacies of colonialism, such as political and economic structures [5], [22], healthcare systems [91], power dynamics [18], [50], [63], [78], [93], [99], behaviours [100] and partnership inequities [21], [98], [99], [101] continue to permeate across, and be entrenched within, global health.

The colonial legacy has contributed to the gap in health outcomes [69] and life expectancy [102] between high and low income countries. One enduring problem is that the default patient in the healthcare field is often considered to be a white male, therefore decolonising symptoms, signs and investigations is an important part of why decolonising global health is needed. For instance, white European biochemical and visual norms used universally can increase the risk of misdiagnosis across populations [100].

As such, the argument for decolonising global health comes from the imperative to hold actors, funders and enablers in global health programmes accountable for their (in)action [52], and address ongoing political manipulation, hypocrisy and distrust [103] in order to redefine roles in international partnership [68], and redress injustices and improve health equity [70], [104].

It is also interesting to note the timing of the proliferation of literature on decolonising global health. Two significant events could be associated with this. Firstly, the Black Lives Matter movement in response to a number of highly publicised cases of US police officers shooting unarmed Black people led to an increase in global awareness of social justice with people working in diverse occupations, ranging from health to education [105] to engineering [106] sparking decolonising movements for those sectors. Secondly, a number of authors emphasised the important role the COVID-19 pandemic had in highlighting inequities in global health outcomes, partnerships and the delivery of global health programmes, especially in terms of personal protective equipment, medical treatment and vaccinations [107], therefore underlining the need to decolonise global health [6], [78], [88], [108].

### 3.6 Approaches to decolonising global health

Frameworks, as a means of understanding the need to decolonise global health and as an approach to put a decolonising approach into action were identified in a number of the papers in our search. Amongst the papers included in the more recent of our two searches (December 2024), we noted a greater emphasis on the application and action in the frameworks and approaches identified than amongst the papers identified earlier [35], [47], [48], [109], [110], [111]. None of these drew directly from decolonial, anticolonial, antiracism, or critical race theory frameworks.

The frameworks and approaches set out in the papers we included share overlapping themes and approaches. These include utilising an epistemic injustice framework, a focus on ‘true’ partnership working, rethinking the governance of global health, considering individual duties, alongside actions that cut across all these approaches.

i) Epistemic injustice

The epistemic injustice framework approach focuses on countering testimonial injustice, specifically where local expertise is excluded from research and local knowledge production is deemed as illegitimate or lesser. It also focuses on countering interpretive injustice by using local interpretive tools and ensuring research aims are not solely aligned to the dominant western audience [54], [70].

The importance of local knowledge and context is consistently highlighted [78]. Avoiding judgement based on foreign cultural norms and using indigenous driven leadership [29], [48], [87], [91], [112], [113] are key to this. Local expert inclusion should be appreciated [35], [47], [59], [60], [78] not just be seen as a tick box [32] and there should be reciprocal flow of knowledge[16].

This can be done through mentoring, investing in researchers and agreeing priorities locally [31]. Building up local expertise and supporting existing programmes in LMICs rather than designing programmes around the needs of western students is a way to shift the locus of control [34], [113], [114]. Likewise, medical journals should diversify their boards [31] as the gatekeepers of global health knowledge. Local indigenous knowledge can and should be re-legitimised [40], [90], [112] through reflection on the terminology used when discussing global health challenges [20], [115] and making room for alternate epistemic standpoints [17], [116]. There should also be equity in data collection, analysis, usage and storage [29], [54] to ensure all partners have equitable access, as well as the inclusion of representative participants and researchers in clinical trials, which otherwise leads to a dependence upon clinical guidelines from HIC settings [60].

Empirical evidence backing epistemic reform includes qualitative investigations into health communication [36], higher education curricula [71], [72], and indigenous health research [3], which collectively illustrate how Eurocentric knowledge systems systematically undervalue local expertise.

ii) Partnership working

Partnership working is related to epistemic injustice approaches and forms a fundamental part of how the literature suggests approaching decolonising global health, particularly in academic partnerships. Beyond the predominantly theoretical discourse, a subset of qualitative and mixed-methods studies in our sample evaluated partnership dynamics in real-world settings. These studies consistently document that despite commitments to equity, resource control and decision-making authority remain concentrated in HIC institutions throughout the project lifecycle [58], [80], [97], [117]. Partners should have shared decision making with strategic priorities and implementation driven by those trusted by affected groups [8], [97].

Fundamentally, the locus of control should sit with local institutions rather than ‘expert’ foreign partners [47], [118], abiding by the principle of *“no research about us, without us”* [23], [117], the motto of the community engagement programme in Brazil, Ethiopia, and Sri Lanka explored in the qualitative field study by Polidano et al. [117] Through partnership working, communities and global health practitioners based in LMICs should be empowered to shape global health interventions [34], [80], [113], [119]. This is a contrast to global health historically, where global health expertise has been concentrated in legacy powers [5] and concerns about the current structure of global health reflecting ‘feudal power’ [93]. This is because funding is suggested to favour HICs with no direct funding to LMICs, instead using ‘feudal intermediaries’ thereby retaining power within the hierarchy of the feudal structure. Dako et al. [35] and Kumar et al. [109] call for research funding to be more equitably distributed, for greater funder accountability and an end to the donor-driven model. This would entail that funding always includes institutions rather than be structured around local partners being used as sub-grantees, and treatment of researchers should be the same regardless of their origin [86]. Nassiri-Ansari et al. [11] concur that greater South-South cooperation in the form of funding and new funding models that favour multilateralism over bilateral arrangements, operate without stipulations, and respond to locally identified needs would *“shift power from donors to the doers”*.

iii) Governance of global health

Many approaches to decolonising global health also focus on rethinking the governing structures of global health. This can include rethinking and restructuring governance relationships that shape decisions [6], [23], [55], [87], [119] rigorous analysis of power asymmetries [120] and not arbitrary movements [22], and rebuilding research infrastructure from the ground up [21]. Kwete et al. [88] argue that addressing structural issues in global health requires decolonising the political economy first.

One critical area of focus is the interaction between global health and the aid industry. To decolonise global health it is suggested that this needs to start with debt cancellation and non-earmarked budget support [53] together with the fair allocation of resources [75] to ensure both greater accountability from donors and funders and to empower LMICs to set their own priorities.

It is also suggested that global health education and workforce policies are central to addressing governance-related inequalities. Reforms needed include broadening methods of education and research, changing teaching for western students [34], [78], [114] changing the location of where global health education happens [65] and reducing the reliance of HICs health service and research institutions on talent from LMICs [60], stopping the *“brain drain”* of health workers from LMICs [60], and reimagining global health as social medicine [121].

In academia, decolonising global health can be promoted through diversity in authorship, integrating local contexts and knowledge systems into research and publications [99], [122], ensuring papers have multilingual abstracts [20] and including study of the place itself as part of the work [95].

iv) Actions for Individuals

Though much of the focus on approaches to decolonising global health is systemic and structural, some literature does highlight an individual's power to change things. Practitioners are asked to reflect and question institutional and structural practices and not to act beyond their expertise [114]. Those engaging in decolonising global health initiatives should be rewarded for their effort and risk in speaking out [8] and not accepting the status quo [114]. This can contribute to building a culture of reflexivity by sparking dialogue in institutions, with the aim of resulting in collective action [123].

It is clear that with such varying systemic, structural and individual issues, decolonising global health will not happen quickly or smoothly. Thus far there has been a highlighting of the need for change and this is a step in the right direction. *“When we make visible this implicit ideological function that the global health field performs, we can see new directions, new ideas and new allies for collective action that are otherwise kept unimaginable”* [124]. This is aspirational but also requires a pathway to success with action-guiding steps. These could include a commitment to a clear list of reforms to address decolonising global health with metrics to track the progress of these reforms [17], [98] to ensure any action is accountable.

### 3.7 Barriers to decolonising global health

Implementation of decolonising global health faces complex barriers rooted in both historical legacies and contemporary dynamics.

One critical hurdle is the neglect, at the individual level, to *“emancipate and decolonise our own minds (from the colonial conditionings of our education)”*, a fundamental step often overlooked in the discourse. According to Abimbola et al. [64], colonialism sought to infiltrate and manipulate fundamental human values, with the colonial classroom serving as a means of psychological domination in Africa and other regions. They suggest that the lingering effects of colonial education policies have instilled a sense of inferiority in many individuals, highlighting the urgent need to dismantle this mindset and reclaim autonomy over our perceptions and judgments. Failure to address this aspect impedes progress by perpetuating entrenched ideologies and power imbalances.

Araújo et al. [59], Farag [125] and Gedela et al. [126] believe the undervaluing of science produced in LMICs, particularly by global health leaders, is both an area that the epistemic injustice approach to decolonising global health should seek to address, and an ongoing barrier to decolonising global health. The persistence of such attitudes risks reinforcing disparities and hindering the integration of context-specific solutions into global health initiatives.

Moreover, a dominant perspective in the literature suggests that decolonisation efforts often fall short in addressing what is perceived as the root cause of coloniality: white supremacy. According to Binagwaho et al. [65], the global influence of white supremacy, rooted in geographic distinctions and skin colour disparities, pervades various societal domains worldwide. Addressing its presence in global health education is crucial, as its elimination could profoundly benefit the health and welfare of marginalized populations globally. Binagwaho et al. [65] and Finkel et al. [99] argue that the existing global health discourse inadequately confronts the ongoing manifestation of white supremacy, particularly evident in leadership inequities where white men from HICs dominate key positions and decision-making power is concentrated in HICs. This lack of diversity in senior roles is exacerbated by confusion about the meaning of decolonisation as opposed to inclusion and diversity [8].

Yerramilli [53] also argues that the success of the decolonising global health movement may be dependent on much wider global factors related to decolonising the world's political economy. This entails dismantling the deep-seated socio-economic inequities exacerbated by historical colonisation. Yerramilli argues that decolonising global health requires us to challenge our concepts of ‘aid’, as this implies voluntary relief, and ‘sustainability’ as this ignores the ongoing political and economic oppression of developing countries. Reimagining the global political economy alongside financial compensation for countries' historical and ongoing outflows is needed to truly decolonise global health [53] and the overarching influence of the world's political economy all complicate the path to decolonising global health [21], [52]. Finally, Hellowell argues that while the decolonisation agenda has the potential to stimulate a much needed redistribution of decision-making power in global health, it also has the potential to undermine confidence in scientific knowledge, accentuating intergroup and international antagonisms and curtailing the opportunities for redistribution in the future [85].

### 3.8 Perspectives from low- and middle-income economies

As the importance, and lack, of LMIC voices emerged as a theme in the narrative analysis, we decided to highlight the perspectives of LMIC authors in this scoping review. Of the 129 papers included in our review, 16 papers (12%) were written by an author or authors exclusively from LMICs. Forty-five papers (35%) named authors from an LMIC alongside authors from HICs.

The findings suggest that LMIC originating papers conceptualise decolonisation and propose broadly similar ideas when compared to papers written by authors from HICs. This observation raises important questions about why this might be. Is it perhaps a manifestation of the lingering effects of colonial education and mindset raised by Abimbola et al. [120] Mogaka et al. [21] posit that researchers from LMICs are frequently educated and operating within or striving towards the global health frameworks and norms established by HICs. It may be a question of methodology; the process by which we identified authors from LMICs, by only referring to papers where authors worked for organisations based in LMICs, may miss or exclude those authors from LMICs who may now reside in HICs [110], [127]. It is also plausible that the search terms used have identified publications by authors who share similar perspectives. And notably, these papers have not been reviewed in isolation and are also embedded within the narrative set out above. In addition, restricting our review to English-language literature likely biased our sample towards LMIC scholars embedded within Anglophone academic networks, while omitting non-English decolonial discourses that might offer distinct conceptual viewpoints.

A particularly strong concern from LMIC authors discussed the disparity of funding and expertise which is perpetuated by the current structures and processes operating across global health [21], [59], [65], [117], [123]. There is a clear demand for parity in partnerships [21], [41], [65] with local expertise and leadership recognised and utilised [41], [101], [117], [123], in a real rather than tokenistic manner [92], [110], [128]. Three papers describe the importance of human factors in decolonisation [88], [123], [129], highlighting that change will not happen without the self-reflection of global health practitioners. Sharma and Sam-Agudu [130] emphasise the particular need for practitioners from the “Global South” to take action to decolonise global health, as the biggest stakeholders and those who stand to benefit the most from decolonising global health. Ssyennyonjo et al. present the decolonising agenda as an opportunity that could be leveraged in order to achieve strategic aspirations defined by Africans themselves [131], through Agenda 2063, a strategic document created by the African Union [132].

## 4 Discussion

In this paper we have reviewed existing literature around the topic of decolonising global health and reported the range of different discussions and views. We have found that the decolonising global health movement broadly attempts to define what decolonising global health is, explore power imbalances in current global health structures, and propose ways to address these imbalances. Our search showed that the majority of the literature about decolonising global health is in the form of commentary or opinion pieces, with authors based in HICs dominating, especially from settler colonial contexts such as the USA, Canada and Australia (80 papers), although it is difficult to accurately assess every author's background. As also reported by Rees et al. [127], although there has been an expansion of publications regarding decolonising global health, the narrative on these topics has primarily been told by authors affiliated with HICs.

Much of the literature describes the historical origins of global health through colonial medicine and attempts to define decolonising global health in order to frame the discussion on decolonising global health. This then helps define why the decolonising global health movement is needed as a means to address epistemic injustice and reduce health inequalities. Our review suggests that there is no universally accepted definition of decolonising global health, that there is uncertainty over how the term originated and is used to further the interests of HICs, and that there is a question as to whether it is the most appropriate response to the inequities driven by colonialism within global health.

The literature included in our review was generally consistent on approaches to decolonise global health with suggestions including: empowering local communities, increasing opportunities for those in LMICs, addressing epistemic imbalances, moving more decision-making power into LMICs, challenging the assumption of the white, European, male as the default reference point for medical practice, and restructuring global health so that leadership and structures are more representative of the global community.

It is also interesting to note the large number of manuscripts (n = 80) from settler colonialism contexts where there may have been erasure and suppression of indigenous knowledge systems in the past and there may be moves to revitalise indigenous epistemologies. This could have impacted both the quantity of manuscripts from these locations as well as “settler guilt” in calling for decolonising global health. The absence of liberation as a framing or definition for decolonising global health perhaps indicates the HIC gaze of the literature, where authors, such as ourselves, are more concerned with adapting our programming to have a decolonial lens rather than extricating countries from colonial systems.

While there is broadly a consensus across the literature we reviewed regarding the need for change, there is less agreement on the mechanisms and priorities for implementation. We note that frameworks and approaches set out in a number of papers share overlapping themes, reflecting recurring concepts, priorities, and critiques within the field. However, it is important to note that frameworks and checklists are critiqued as reflecting western approaches to knowledge production, particularly when they emphasise standardised methodologies that may not fully account for context-specific realities in LMICs [100], [112].

Papers identified in the most recent of the two searches of the literature which we undertook more frequently presented actionable next steps for decolonising global health. However, where steps are set out (using data and metrics [17], subsidiarity and shared decision making [133], overhauling the global health industry [134]) it is often not clear how these should be coordinated and by whom. This is potentially an outcome of the nascent nature of discussions on how to decolonise global health and further work on this may be forthcoming.

There are clear barriers to decolonising global health that are described within the literature. There is a recognition that much of the imbalance stems from the donor-recipient relationship as a consequence of how aid works in our current global geopolitical system. Indeed, some commentators highlighted that global health in its current format is part of the problem, entrenching imbalances and power dynamics through the use of aid as soft power. Some authors suggested that the structural changes needed are wider than the global health system and extend to looking at the global neo-liberal capitalist system as the root of these inequalities. Despite the prevailing call for structural changes, there appears to have been limited efforts to address these in major global health institutions with only a few exploring how the changes in practice suggested in the literature may be implemented. None of the papers identified in our review reported the results of changes or interventions to implement any such changes in practice. This may be due to a tension between the structural changes called for in global health relying on change to be driven by those leading those very global health institutions that are acknowledged as part of the problem. Indeed, the question remains, who is best placed to drive forward the structural changes in order to make global health more equitable.

Additionally, we found that, although commentary or opinion pieces made up the majority of the literature, the 34 non-commentary studies included in this review provide important empirical evidence documenting how colonial power dynamics play out in practice. Qualitative and mixed-methods investigations demonstrate how colonial power dynamics manifest in daily practice, for instance, revealing power asymmetries across the global health project cycle [97], documenting gatekeeping in academic publishing [127], and illustrating donor-driven priorities in field implementation [117]. Grounding the development and interpretation of decolonial frameworks in the findings from empirical studies will be an important step in moving beyond the theoretical to inform decolonial practice.

In terms of specific actions or principles to decolonise global health, attempting to summarise the papers appraised in this review, we propose the following as a starting point to further the discussion. These are orientated by the same conceptual classification we saw in the literature on decolonising global health.

### 4.1 Recommended approaches and next steps for decolonising global health

Drawing from the findings of this review, below we propose a set of approaches and next steps for decolonising global health, focussing on epistemic justice, partnership working, the structure of global health, and individual duty.

Epistemic justice:

1. While it is useful to have a shared terminology and understanding in order to promote good practice, this does not have to be framed as ‘decolonising global health’. Alternative terms from LMIC global health actors should be considered.
2. To promote and value knowledge systems beyond those traditionally centred in HICs, academic tools, such as quality assurance frameworks, should be re-engineered to ensure that they reflect a plurality of epistemic viewpoints.
3. Global health research, funding, educational and leadership opportunities should be prioritised for global health actors from LMICs to challenge the historical marginalisation of local knowledge and enhance decision-making rooted in specific cultural and political contexts.
4. To confront the colonial legacy of privileging certain ways of knowing, global health actors, institutions and funders should acknowledge diverse forms of expertise and knowledge exists. To include lived experience, local, cultural, political and the traditional knowledge of individuals and communities.

Partnership working:

1. Global health actors should have a duty to implement and evaluate shared decision-making frameworks and processes in global health collaborations.
2. Global health actors should adopt a minimum standard to work towards equitable partnerships, ensuring fair access to material and human resources, especially for LMIC partners who have historically been excluded.
3. Evaluations of global partnerships should include explicit assessments of power asymmetries and structural inequities, which are of central importance in decolonising global health.

Structure of global health:

1. There is a need to generate interventions and metrics to support the decolonisation of global health. These should answer questions such as *‘How do we know how institutions are currently performing?’* or ‘*How do we know that an intervention (*e.g. *decolonising a learning curriculum) generates the intended outcome and does not accentuate harm.’* There is a need for methodological inquiry and deliberation to think about what metrics would be valid, contextually relevant and not rooted solely in the evaluative standards of HICs.
2. Financial aid flows from HICs to LMICs should be targeted towards LMIC researchers or LMIC-led collaborations to shift power and decision-making authority.
3. There should be increased diversity and active representation from across LMICs in global health leadership positions, as an important step in dismantling existing power imbalances and ensuring that global health leadership reflects the communities it serves
4. Where organisations commit to decolonising global health, system-wide, organisational and individual accountability mechanisms should be embedded to ensure recommendations are addressed and actions are evaluated.

Actions for Individuals:

1. Reflective practice in global health should be matched with peer dialogue and insight driven action to better support conditions within organisations to support efforts to decolonise.
2. Global Health actors or those working in or researching global health topics should advocate for decolonising global health to make visible what is at stake, including whose health is prioritised, whose knowledge is legitimised, and who benefits from global health systems.

These recommendations serve, not as an end point, but as a pragmatic starting point for furthering the discussion on how decolonising global health could be driven forward with a view to achieving more equitable outcomes and impact across the sphere of global health. We therefore welcome discussion and critique of these, with the hope that compiling this disparate literature aids in revealing a clearer path forward for this critically important movement.

### 4.2 Strengths and limitations of this review

Despite the recent expansion of scholarship on decolonising global health, existing syntheses remain narrow or niche-specific. This scoping review addresses this gap by providing a broad-based collation across 129 peer-reviewed and grey literature sources. The review demonstrates that despite the dominance of theoretical commentaries (n = 95), over a quarter of the evidence base (n = 34, 26.4%) comprises empirical qualitative, mixed-methods, and case studies that document how colonial power dynamics operate in daily practice, specifically through project-cycle power asymmetries, publishing gatekeeping, and donor-driven funding priorities. Additionally, by mapping author affiliations and first-authorship hierarchies, this study demonstrates that HIC institutions continue to hold lead authorship in 69% of HIC–LMIC collaborative papers within the decolonisation literature itself. Finally, by moving beyond theoretical critique, this review pulls together themes from across the literature into a set of recommendations and actionable next steps for decolonising global health, focussed on epistemic justice, partnership working, structural governance, and individual duty.

However, alongside these strengths a number of methodological limitations should be acknowledged. First is the exclusive focus on English-language, written academic and grey literature. This restriction will have excluded valuable decolonial scholarship and perspectives published in other languages, such as Spanish, French, Portuguese, and indigenous languages, which are central to decolonial discourse in Latin America, Francophone and Lusophone Africa, and Asia. Additionally, the focus in this review on traditional written literature excludes non-academic modes of knowledge transmission, such as community storytelling, oral histories, podcasts, and digital activism. As reflected in our positionality statement, these boundaries mirror broader structural gatekeeping within Anglophone academic publishing, which likely contributed to the high proportion of HIC affiliated authors identified in our included papers. Future review work should aim to incorporate multilingual teams and non-written work to capture a truly representative global discourse.

A further limitation is that the inclusion criteria focused on papers discussing ‘decolonising global health’ as the primary way to identify papers for inclusion in our review. Focussing on these search terms may have biased the papers we identified and the views expressed in those papers, as other terms used for the same concept may not have been captured. As we have explored, criticisms of the decolonising global health movement include the idea that this is a conversation that is mostly being driven by HICs and may now be being used as a buzzword to demonstrate equity, justify institutional existence in the global health space to funders and political leaders and thereby perpetuate existing power and knowledge structures. It may also be that similar conversations are occurring in the literature without using the specific term ‘decolonising global health’ that we have not therefore captured in this review.

Finally, two additional methodological considerations should be noted regarding our data synthesis. First, determining author origin solely by primary institutional affiliation at publication serves as an imperfect proxy for geographic representation. This approach does not fully capture LMIC scholars based at HIC institutions, diaspora researchers, or authors holding dual affiliations. Second, while omitting formal quality appraisal aligns with the standard scoping review methodology to map the breadth of an evolving field, the evidence base retrieved consists overwhelmingly of theoretical commentary and opinion pieces (n = 95). None of the included studies evaluated or measured outcomes of implemented decolonial interventions in practice. Consequently, our synthesis reflects proposed conceptual frameworks and rhetorical calls to action rather than tested operational models.

### 4.3 Conclusion

Although the literature on decolonising global health has expanded rapidly in recent years, this scoping review demonstrates that the field remains constrained by uncertainty over definitions and concepts, an over-representation of HIC author affiliations, and a limited body of evaluated implementation models. Despite these limitations, there is clear consensus across the literature that current global health structures continue to perpetuate historically rooted power asymmetries. Addressing these inequities requires moving beyond theorising or the superficial adoption of decolonial language, which risks becoming a set of academic "buzzwords" that signal equity without fundamentally shifting decision-making power or resource control.

Achieving structural parity does not depend on adopting Western-defined terminology, and supporting LMIC-led work to develop a consensus on preferred frameworks will be essential. Alternative LMIC-led models, such as the Africa Centres for Disease Control and Prevention's *'New Public Health Order for Africa'*, demonstrate how centring local priorities, community leadership, and rebalanced donor-donee relationships can be operationalised without using or preferring 'decolonisation' terminology [135]. Strengthening regional health governance institutions, including the Africa CDC and the ASEAN Centre for Public Health Emergencies and Emerging Diseases [136], provides a tangible, practical pathway to shift leadership to local actors and ensure equitable access to critical interventions, such as vaccines.

The recommended approaches and next steps for global health actors proposed in this review, focussing on epistemic justice, equitable partnerships, structural governance, and individual reflexivity, offer a pragmatic starting point for global health actors and institutions. By confronting colonial legacies, shifting power to regional and local institutions, and embedding accountability into global health governance, the discipline can begin to move from rhetoric to action and realise its foundational commitment to health equity and social justice.

## Author contributions

Daniel E Stewart: Conceptualisation, data curation, formal analysis, investigation, visualisation, project administration, writing original draft, review and editing Tiruneh Amsalu: Conceptualisation, formal analysis, investigation, writing original draft, review and editing Ellie Fairfoot: Conceptualisation, data curation, formal analysis, investigation, visualisation, writing original draft, review and editing David Keen: Conceptualisation, data curation, formal analysis, investigation, writing original draft, review and editing Jess Keenan: Data curation, formal analysis, investigation Frances Butcher: Writing original draft, review and editing Kevin Miles: Conceptualisation, data curation, formal analysis, investigation, supervision, writing original draft, review and editing Ahmed Razavi: Conceptualisation, data curation, formal analysis, investigation, supervision, writing original draft, review and editing.

## Ethical statement

As this study was a scoping review, based exclusively on the synthesis of previously published literature, formal ethical approval was not required.

## Funding

This research did not receive any specific grant from funding agencies in the public, commercial, or not-for-profit sectors.

## Declaration of competing interest

The authors declare that they have no known competing financial interests or personal relationships that could have appeared to influence the work reported in this paper.
